# Vav Links the T Cell Antigen Receptor to the Actin Cytoskeleton and T Cell Activation Independently of Intrinsic Guanine Nucleotide Exchange Activity

**DOI:** 10.1371/journal.pone.0006599

**Published:** 2009-08-12

**Authors:** Ana V. Miletic, Daniel B. Graham, Kumiko Sakata-Sogawa, Michio Hiroshima, Michael J. Hamann, Saso Cemerski, Tracie Kloeppel, Daniel D. Billadeau, Osami Kanagawa, Makio Tokunaga, Wojciech Swat

**Affiliations:** 1 Department of Pathology and Immunology, Washington University School of Medicine and Siteman Cancer Center, St. Louis, Missouri, United States of America; 2 Research Unit for Single Molecule Immunoimaging, RIKEN Center for Allergy and Immunology, Yokohama, Kanagawa, Japan; 3 Department of Immunology and Division of Oncology Research, Mayo Clinic College of Medicine, Rochester, Minnesota, United States of America; 4 Laboratory for Autoimmune Regulation, RIKEN Center for Allergy and Immunology, Yokohama, Kanagawa, Japan; 5 Structural Biology Center, National Institute of Genetics, The Graduate University for Advanced Studies, Mishima, Shizuoka, Japan; 6 Department of Genetics, The Graduate University for Advanced Studies, Mishima, Shizuoka, Japan; New York University School of Medicine, United States of America

## Abstract

**Background:**

T cell receptor (TCR) engagement leads to formation of signaling microclusters and induction of rapid and dynamic changes in the actin cytoskeleton, although the exact mechanism by which the TCR initiates actin polymerization is incompletely understood. The Vav family of guanine nucleotide exchange factors (GEF) has been implicated in generation of TCR signals and immune synapse formation, however, it is currently not known if Vav's GEF activity is required in T cell activation by the TCR in general, and in actin polymerization downstream of the TCR in particular.

**Methodology/Principal Findings:**

Here, we report that Vav1 assembles into signaling microclusters at TCR contact sites and is critical for TCR-initiated actin polymerization. Surprisingly, Vav1 functions in TCR signaling and Ca^++^ mobilization via a mechanism that does not appear to strictly depend on the intrinsic GEF activity.

**Conclusions/Significance:**

We propose here a model in which Vav functions primarily as a tyrosine phosphorylated linker-protein for TCR activation of T cells. Our results indicate that, contrary to expectations based on previously published studies including from our own laboratory, pharmacological inhibition of Vav1's intrinsic GEF activity may not be an effective strategy for T cell-directed immunosuppressive therapy.

## Introduction

In developing and mature T cells, the T cell receptor (TCR) activates Src family kinases that phosphorylate immunoreceptor tyrosine-based activation motifs (ITAMs) in CD3 and TCRζ proteins, providing docking sites for Syk/ZAP-70 family kinases. Subsequently, the recruitment of the adaptors LAT, GADS, and SLP-76, and enzymes such as Tec family kinases, phosphoinositol-3 kinase (PI3K), and phospholipase Cγ1 (PLCγ1), leads to the generation of the secondary signaling intermediates, 1,4,5-inositol triphosphate (IP_3_) and diacylglycerol (DAG), activating intracellular Ca^++^ and mitogen-activated protein kinases (MAPK) (reviewed in [1], [2]). Together, these events promote the transcription of genes involved in T cell proliferation and differentiation. The engagement of the TCR also leads to rapid and dynamic changes in the T cell actin cytoskeleton that can be visualized by imaging F-actin. In a model of TCR stimulation on a planar surface, F-actin is induced at TCR-surface contact sites, but then spreads circumferentially to the cell periphery driving plasma membrane extensions such as filopodia and lamellipodia [3]. In addition, recent live cell imaging studies using total internal reflection fluorescence microscopy (TIRFM) in combination with stimulatory antibodies or planar bilayers containing peptide:MHC complexes revealed the formation of microclusters of signaling proteins including TCRζ, CD3, ZAP-70, SLP-76 and Vav, suggesting that these structures could be the sites of signal generation [4], [5], [6], [7], [8], [9]. Nevertheless, while the importance of the actin cytoskeleton in lymphocytes has been appreciated for over 30 years, the exact mechanism(s) by which the TCR initiates actin polymerization remains incompletely understood [10].

Several models have been proposed for TCR-initiated actin polymerization (reviewed in [10], [11], [12], [13]). While most studies point to the involvement of WASp/WAVE proteins as the downstream effectors, important differences exist in the proposed mechanisms regarding how the TCR is linked to actin assembly. For example, one model suggests that CD3 chains directly recruit an Nck-WASp complex via Nck SH3 binding to proline-rich sequences in CD3 [14], providing an explanation of how F-actin induction could occur at the TCR independently of ITAM phosphorylation. However, the preponderance of evidence indicates that tyrosine phosphorylation and the recruitment of ZAP-70, SLP-76, and LAT are required for TCR initiation of F-actin assembly, and recent studies suggest that microclusters of these signaling proteins (also termed proto-synapses) can recruit WASp to sites of TCR contacts [6], [15], [16].

In this context, WASp/WAVE-mediated nucleation of actin filaments, through their interaction with the Arp2/3 complex, can be induced by Nck binding independently of Rho GTPases [17], [18]. Alternatively, WASp/WAVE activation can be mediated by Rho GTPases, such as Rac1 and Cdc42, which are activated by guanine nucleotide exchange factors (GEF), including Vav, αPIX, βPIX, and DOCK2 [19], [20], [21], [22], [23], [24]. Vav has been implicated in T cell cytoskeletal regulation based on its Dbl-homology (DH) domain, tyrosine phosphorylation, and recruitment to T cell-APC contacts (reviewed in [25]), although recent studies indicated the importance of Vav in integrin activation and T cell-APC conjugate formation, rather than in F-actin assembly [26], [27]. Thus, while Vav1 also regulates ERM [28] and MTOC polarization [26], no conclusive evidence exists, to date, in support of an essential role for Vav proteins in the TCR initiation of actin polymerization. In this regard, because studies of T cells lacking all three Vav proteins revealed redundancy of Vav1 with other Vavs [29], direct examination of TCR-induced actin polymerization in Vav1/2/3-deficient (Vav^NULL^) T cells should conclusively establish whether or not the Vav family is essential in this process. While Vav is considered a Rho GEF, it is unknown if the intrinsic GEF activity is indeed required for Vav function downstream of the TCR. In this context, disruption of TCR-induced Ca^++^ and MAPK signaling in T cells lacking all Vav proteins (Vav^NULL^) suggests that Vav may function downstream of the TCR as a critical linker rather than exclusively as a Rho GEF [29]. Consistent with such a view, GEF-inactivated Vav has been shown to augment NFAT-dependent transcriptional activation in Jurkat T cells [30]. In addition, Vav contains several tyrosine residues that may be involved in direct binding of SH2 domain-containing proteins [9], [31], [32]. Thus, it is possible that Vav mediates TCR signals independently of its intrinsic GEF activity, however this remains to be tested in T cells lacking all endogenous Vav proteins.

In this report, we address these unresolved issues. Using live-cell imaging, we show that Vav forms signaling microclusters at TCR contact sites, similar to other TCR linker proteins, and demonstrate that the Vav family is critical for TCR initiation of actin polymerization. Surprisingly, the intrinsic GEF activity is dispensable for Vav function in TCR signaling and mobilization of intracellular Ca^++^ fluxes. Here, we propose a model for Vav as a critical linker in TCR-induced activation of T cells.

## Results

### Vav proteins are essential for the initiation of actin polymerization at the TCR

**Figure 1:**
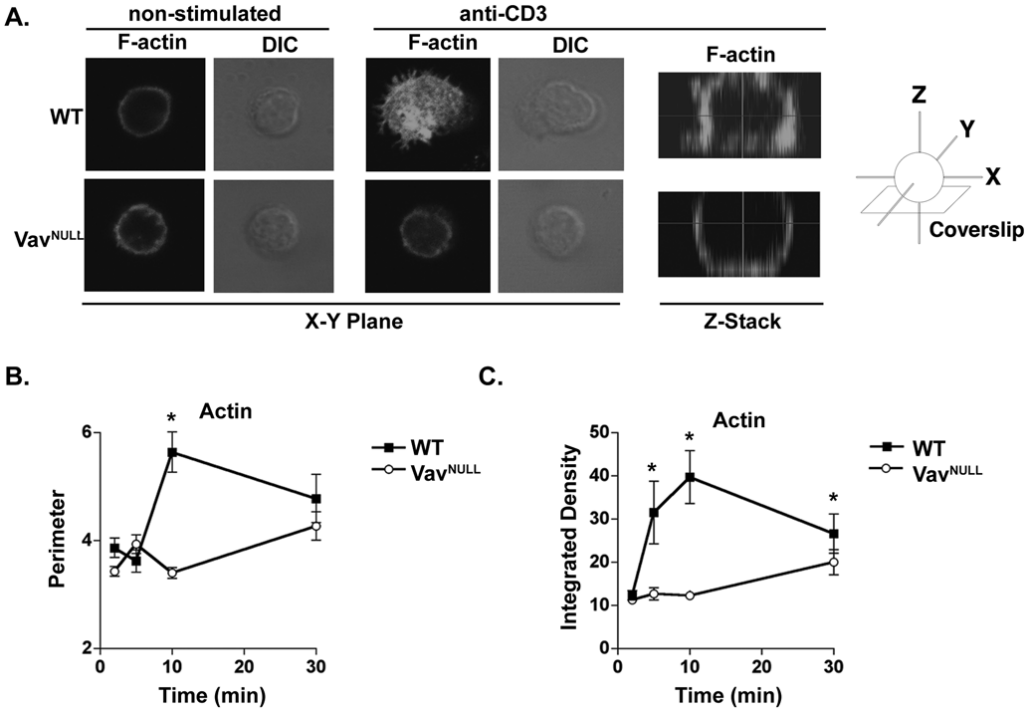
Vav^NULL^ T cells show defective TCR-induced actin cytoskeletal reorganization and cell spreading. (A) Staining of F-actin with Alexa-Fluor-488-phalloidin in WT or Vav^NULL^ T cells stimulated on anti-CD3-coated coverslips and fixed after 10 minutes. Shown are representative images of n≥10 cells. Optical slices in the XY plane depict the cell-coverslip interface, and Z-stacked images depict the entire cell in the XZ plane. (B) Cell perimeter was measured in arbitrary units at the membrane-coverslip interface for T cells stimulated as in (A), data are mean±SD of n≥10 images/time point. (C) The relative concentration of F-actin at the membrane-coverslip contact site measured as integrated density (pixel intensity) in arbitrary units of Alexa-Fluor-488-phalloidin fluorescence within the perimeter of the membrane-coverslip contact site described in (B). Asterisks (*) indicate p<0.05.

In view of the functional redundancy of Vav proteins, we decided to examine if the Vav family is required in TCR-initiated actin polymerization using Vav^NULL^ T cells lacking all 3 Vav proteins [29]. To this end, we first analyzed WT T cells by confocal imaging of F-actin structures at the plane of cell contact with the stimulatory coverslip, visible by DIC microscopy, and then in increments along the Z-axis (Fig. 1A) [3], [4], [6]. Initially, the cell-contact sites appeared round and did not show significant F-actin content beyond a small ring along the circumference of the cell contact. Subsequently, within 2–5 minutes, WT T cells showed dramatic F-actin accumulation throughout the region of coverslip contact and formed filopodia and lamellipodia stretching beyond the circumference of the F-actin ring (Fig. 1A, and data not shown). This process continued for approximately 10 minutes, at which time the cell perimeter (Fig. 1B) and F-actin content (Fig. 1C) reached their maximum. We next analyzed Vav1-deficient (Vav1^−/−^) T cells and found that cell spreading and the induction of F-actin structures were delayed relative to WT (Fig. S1), indicating that Vav1 regulates but is not essential for TCR-induced actin polymerization in this system. In sharp contrast to WT or Vav1^−/−^ T cells, F-actin production and cell spreading of Vav^NULL^ T cells was virtually blocked (Fig. 1A,B,C), resembling non-stimulated cells at all of the time points studied (Fig. 1A). These results show that the Vav family is critical for the initiation of TCR-induced actin polymerization and T cell spreading. Thus, together with the involvement of Vav1 in signaling microclusters [9], these data indicate that Vav may function as a critical linker for TCR-initiated actin polymerization, raising the question of whether or not the intrinsic GEF activity is necessary for its function in this process.

### GEF-inactive Vav1 participates in signaling microclusters and restores TCR function in J.Vav cells and Vav1-deficient T lymphocytes

**Figure 2:**
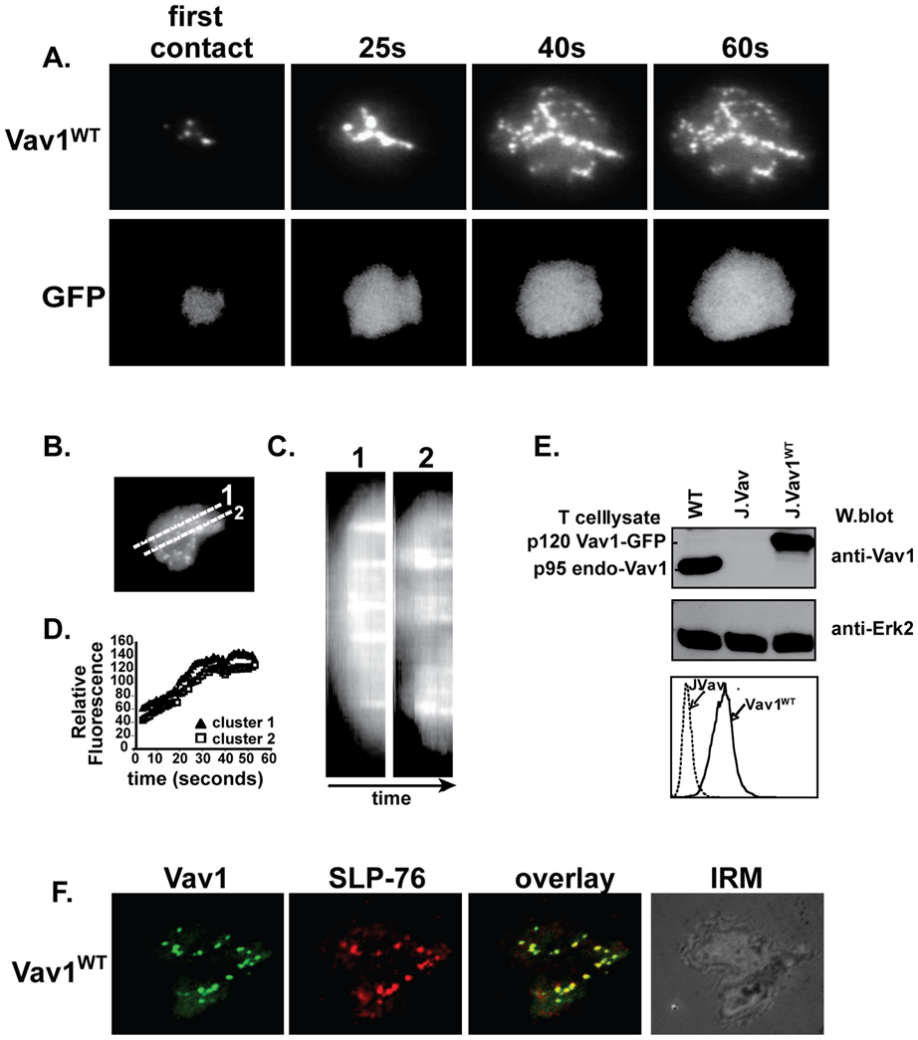
Vav1 forms microclusters in response to TCR stimulation. (A) J.Vav1^WT^ cells were activated on anti-CD3-coated coverslips. Images were obtained in real time using TIRFM (times, above images). (B) Diagonal lines indicate sections of a representative J.Vav1^WT^ cell taken for kymographic analysis. (C) Fluorescence of individual Vav1-GFP microclusters over time (60s) are presented as horizontal “streaks” in kymographs for sections shown in (B). (D) Mean fluorescence intensity over time (60s) of individual Vav1-GFP clusters in stimulated J.Vav1^WT^ cells. Shown are representative images of n = 5. (E) Vav1^WT^-GFP expression. Shown are immunoblots with anti-Vav1 antibodies and FACS of J.Vav1^WT^ cells. (F) Vav1-GFP and SLP-76 microcluster formation in J.Vav1^WT^ cells, activated on anti-CD3-coated coverslips for 2 mins.

Live-cell imaging studies of T cell-planar surface contacts revealed microclusters of signaling proteins that included ZAP-70, LAT, SLP-76, Nck, Grb2, and WASp, which have been implicated in the initiation of T cell activation and actin polymerization at the sites of TCR contacts [4], [5], [6], [7], [33]. Since Vav1 has been implicated in T cell cytoskeleton regulation, we decided to examine its dynamic redistribution in live T cells. To this end, we generated Vav1-deficient Jurkat cells [34] that express Vav1-GFP (J.Vav1^WT^) at the level of endogenous Vav1 in the WT parental Jurkat line [9] (Fig. 2). Such cells were analyzed using stimulatory coverslips and real-time total internal reflection fluorescence microscopy (TIRFM), allowing visualization of Vav1-GFP in the direct vicinity (100–200 nm) of plasma membrane-coverslip contacts. Consistent with our recent report, Vav1-GFP quickly assembled (within 5–10 seconds of initial contact) into microclusters at the cell-coverslip interface (Fig. 2A) [9]. Notably, kymographic analyses of microcluster fluorescence intensity over time, indicate that Vav1-GFP microclusters are stable (Fig. 2B–D), and Vav1 showed little, if any, lateral diffusion as indicated by laser-bleaching (data not shown). Control experiments using J.Vav cells expressing GFP-only (GFP), or J.Vav1^WT^ cells incubated on coverslips with irrelevant antibody or poly-L-lysine showed no significant microcluster formation (Fig. S2 and data not shown). To extend these initial observations, we used confocal imaging and found that TCR-induced Vav1-GFP microclusters colocalized with SLP-76 microclusters (Fig. 2F and Fig. S3). Thus, given that the redistribution pattern of Vav was reminiscent of other signaling molecules implicated in microcluster formation [4], and that Vav colocalized with SLP-76, these data suggest that Vav could be involved at the sites of initial TCR-induced actin polymerization, which is consistent with our finding that Vav is required for generation of F-actin and cell spreading (Fig. 1).

**Figure 3:**
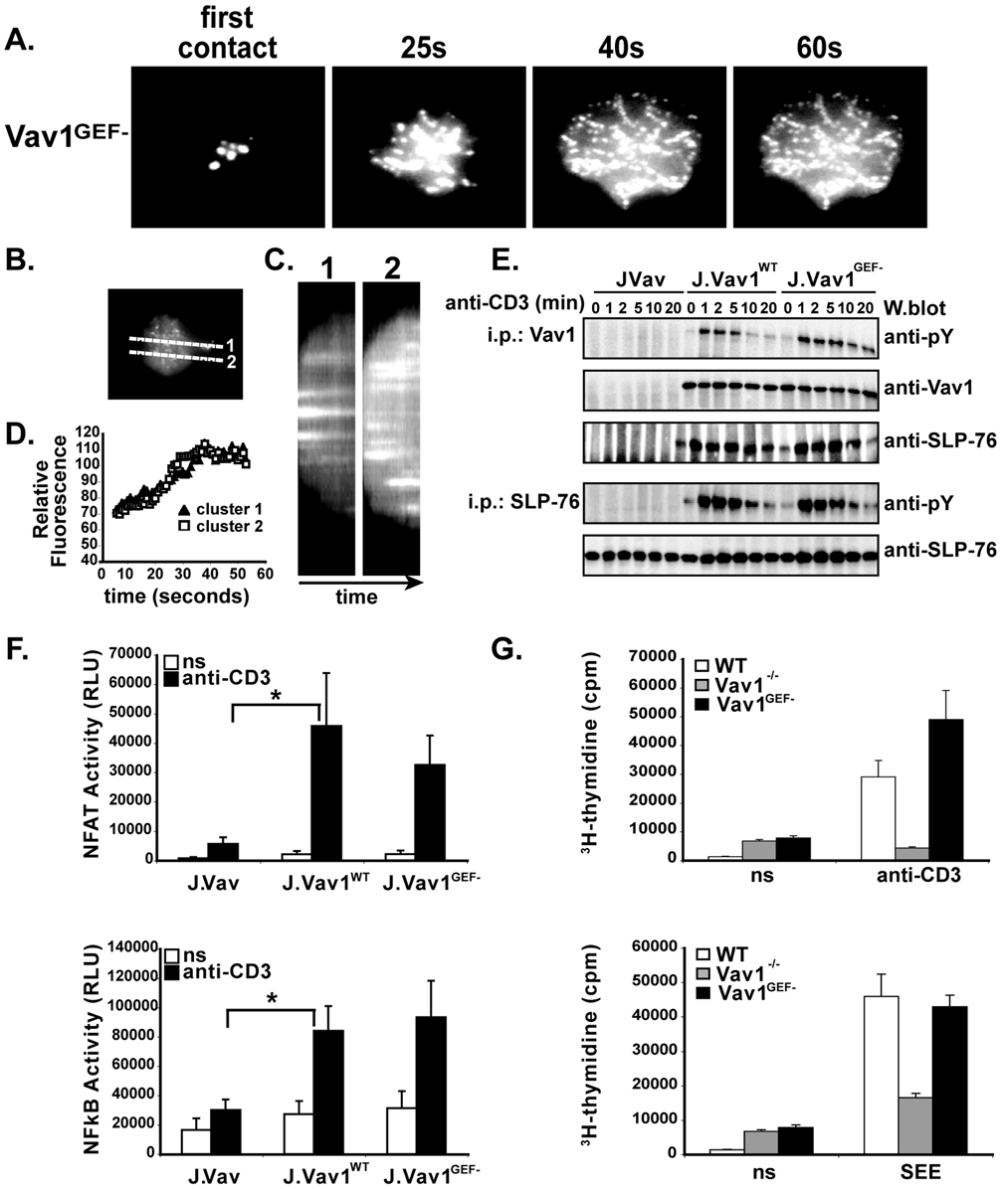
Vav1^GEF−^ forms microclusters and supports TCR-induced transcription and proliferation. (A) J.Vav1^GEF−^ cells were plated on anti-CD3-coated coverslips. Images were obtained in real time using TIRFM (times, above images). (B) Diagonal lines indicate sections of a representative J.Vav1^GEF−^ cell used for kymographic analysis. (C) Fluorescence of individual Vav1^GEF−^ microclusters over time (60s) is presented as horizontal “streaks” in kymographs for sections shown in (B). (D) Mean fluorescence intensity over time of individual Vav1^GEF−^ clusters, shown are representative images of n = 5. (E) Tyrosine phosphorylation of Vav1 or SLP-76 immunoprecipitated from anti-CD3-stimulated J.Vav1^WT^ and J.Vav1^GEF−^ cells, visualized by immunoblotting with anti-phospho-tyrosine antibodies. Binding of SLP-76 to Vav1, determined by reprobing blots with anti-SLP-76 antibodies. ns = non-stimulated. (F) NFAT or NFκB luciferase reporter assays of anti-CD3-activated J.Vav, J.Vav1^WT^ and J.Vav1^GEF−^ cells, data are mean±SD n>5 experiments. (G) Proliferation of WT, Vav1^−/−^ or GFP^+^ Vav1^GEF−^ T cells generated by HSCC reconstitution, as indicated, measured at 48 hr by ^3^H-thymidine incorporation, n = 2. Asterisks (*) indicate p<0.05.

To determine if the intrinsic GEF activity of Vav1 is required for its function in TCR signaling, we first generated J.Vav cells expressing Vav1 protein with a previously characterized GEF loss-of-function mutation L278Q (corresponding to L213Q in onco-Vav), fused to GFP (J.Vav1^GEF−^) ([9], [22], [35], [36], [37] and Fig. S4). We first examined such J.Vav1^GEF−^ cells by TIRFM, as in experiments described in Fig. 2, and found that, similar to Vav1^WT^, Vav1^GEF−^ generated stable microclusters at the T cell-stimulatory coverslip interface (Fig. 3A–D). Moreover, similar to Vav1^WT^, Vav1^GEF−^ microclusters colocalized with TCR-induced SLP-76 microclusters (Fig. S3). In addition, tyrosine phosphorylation and SLP-76 binding of Vav1^GEF−^ in response to TCR stimulation showed no discernible differences from Vav1^WT^ (Fig. 3E). Thus, neither the pattern of Vav1 redistribution, nor its tyrosine phosphorylation and SLP-76 binding, appear to be affected by the loss of intrinsic GEF activity (Fig. 3A–E).

To determine if Vav GEF activity is required for TCR induction of NFAT and NFκB, we used J.Vav1^WT^ and J.Vav1^GEF−^ cells transfected with NFAT or NFκB luciferase reporter-gene constructs and analyzed luciferase activity upon stimulation with anti-CD3 antibodies (Fig. 3F). As expected, such treatment led to a strong induction of both NFAT- and NFκB-dependent luciferase activity in J.Vav1^WT^ T cells. Notably, J.Vav1^GEF−^ cells showed no statistically significant differences in activity in this assay as compared to J.Vav1^WT^ (Fig. 3F) and responded similarly to PMA and Ionomycin (Fig. S5). These experiments suggest that, even in the absence of endogenous Vav1, a GEF-inactive Vav1 is capable of rescuing TCR-induced NFAT- and NFκB-dependent transcriptional activation. These observations are consistent with previous studies showing GEF-independent effects of Vav in this pathway [30]. Strikingly, however, the same GEF-inactivating mutation completely abolished the ability of Vav to activate NADPH-oxidase in myeloid cells ([9], [36], [37], and our unpublished observations). Thus, it appears that in contrast to the TCR signaling pathway, in myeloid cells Vav GEF activity is critical for its function in regulating the NADPH oxidase complex.

Since signaling properties of Jurkat T cells differ in some aspects from those of primary T cells, for example due to PTEN deficiency, we decided to examine the requirement for Vav1 GEF activity in primary T lymphocytes. In this regard, while anti-CD3- or superantigen SEE-induced proliferation of Vav1^−/−^ T lymphocytes was diminished, as expected based on previously published studies [38], [39], [40], expression of retrovirally-encoded Vav1^GEF−^ protein in primary Vav1^−/−^ T lymphocytes restored their proliferative capacity, as compared to Vav1^WT^ T cells (Fig. 3G). In addition, TCR-mediated Ca^++^ signaling, which is defective in Vav1^−/−^ T cells, was restored in Vav1^GEF−^ cells (data not shown).

Taken together, these results suggest that the intrinsic GEF activity is dispensable for Vav1 function in J.Vav cells and in Vav1^−/−^ T cells. However, because neither J.Vav cells nor Vav1^−/−^ T lymphocytes show appreciable defects in TCR-induced actin polymerization (Fig. S1 and data not shown), we reasoned that the requirement for GEF activity must be conclusively addressed in T cells in the Vav^NULL^ background.

### Expression of Vav1^GEF−^ restores T cell development in Vav^NULL^ mice

**Figure 4:**
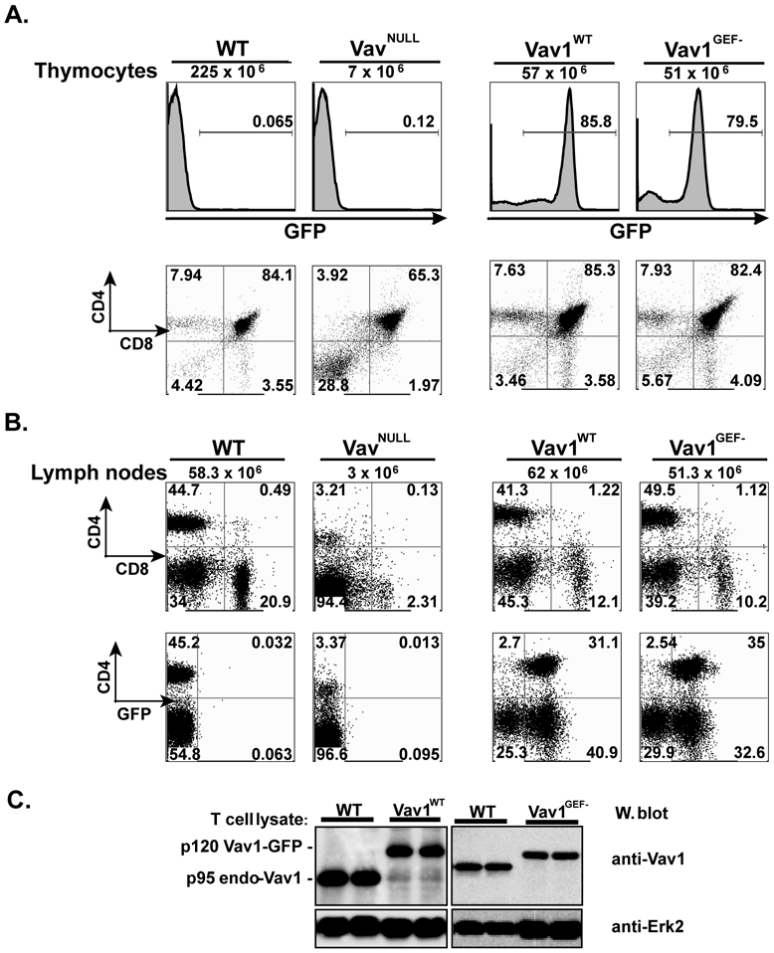
Expression of Vav1^GEF−^ restores Vav^NULL^ T cell development. (A) Flow cytometric analyses of thymocytes from WT, Vav^NULL^, Vav1^WT^ and Vav1^GEF−^ mice. The bottom panel is GFP^+^-gated, shown is one representative of n>5 mice. (B) Flow cytometric analyses of WT, Vav^NULL^, Vav1^WT^, or Vav1^GEF−^ lymph nodes as in (A). The top panel is GFP^+^-gated, shown is one representative of n>5 mice. (C) Expression of Vav1^WT^ and Vav1^GEF−^ proteins in T cell lysates, visualized by immunoblotting with anti-Vav1 antibodies. Protein loading was verified by reprobing blots with antibodies to Erk2.

To address the requirement of Vav GEF activity, without the complicating issue of compensatory effects of endogenous Vav proteins, we decided to generate T cells that express Vav1^GEF−^ in the absence of any other Vav protein. In this regard, we first examined if Vav1^GEF−^ protein could, by itself, support Vav^NULL^ T cell development. To this end, we developed a Vav^NULL^-hematopoietic stem cell complementation (HSCC) approach and, as a validation of this approach, showed that Vav1^WT^-GFP expression rescued Vav^NULL^ T cell development (Fig. 4A,B). Thus, while Vav^NULL^ mice showed severely reduced populations of both developing and mature T cells [29], Vav1^WT^ chimera mice developed populations of thymocytes and peripheral T lymphocytes similar to WT mice (Fig. 4A,B), although the total number of thymocytes generated in such RAG-chimera is typically somewhat lower, as compared to WT (Fig. 4A and data not shown). Thus, having established that the introduction of Vav1^WT^ rescues development of Vav^NULL^ T cells, we next examined the effects of Vav1^GEF−^ in this same assay. Strikingly, both numbers and percentages of thymocytes and peripheral T cell subsets in Vav1^WT^ and Vav1^GEF−^ mice were similar (Fig. 4A,B). Importantly, the levels of expression of Vav1^WT^ and Vav1^GEF−^ proteins were virtually equal to that of endogenous Vav1 (Fig. 4C). Also, similar to Vav1^WT^, a majority of Vav1^GEF−^ thymocytes and peripheral T cells were GFP^+^ (Fig. 4A,B), and these GFP+ cells contained the mutated Vav1^GEF−^, as confirmed by direct sequencing of genomic DNA from purified peripheral T cells (data not shown). Together, these results show that GEF-inactive Vav1 is capable of restoring development of T cells lacking all endogenous Vav family proteins. We conclude from these experiments that Vav GEF activity is not essential in T cell development.

### Expression of Vav1^GEF−^ rescues Vav^NULL^ T cell proliferation and cytokine production

**Figure 5:**
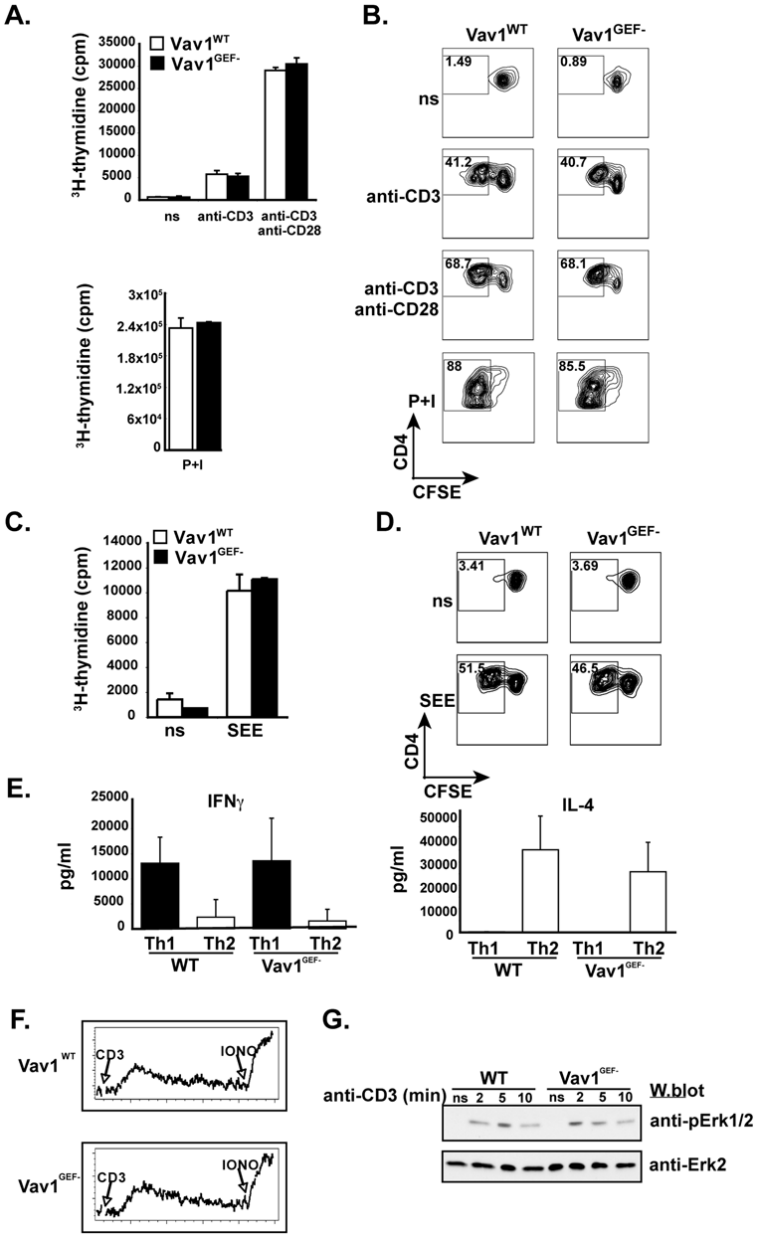
Vav1 GEF activity is not required for T cell function. (A) Proliferation of Vav1^WT^ or Vav1^GEF−^ T cells stimulated with anti-CD3 +/− anti-CD28 antibodies, measured by ^3^H-thymidine incorporation at 48 hours, n>3. P+I = PMA and ionomycin. (B) As in (A), with CFSE dye dilution at 72 hours, n>3. (C) Proliferation of Vav1^WT^ or Vav1^GEF−^ T cells stimulated with SEE-pulsed APCs, measured by ^3^H-thymidine incorporation at 48 hours, n>3. (D) As in (C), with CFSE dye dilution at 72 hours, n>3. (E) Supernatant cytokines from T cells cultured under Th1 or Th2 polarizing conditions were analyzed by ELISA, n>4. (F) Ca^++^ mobilization by CD4^+^ T cells stimulated with anti-CD3 antibodies, n = 5. (G) Erk1/2 activation in T cells activated with anti-CD3 antibodies for indicated time points, visualized by immunoblotting with antibodies against active Erk1/2. Protein loading was verified by reprobing blots with anti-Erk2 antibodies.

Although the Vav family is necessary for T cell proliferative responses [29], [38], [39], [40], the requirement for Vav GEF activity is not known. To address this issue, Vav1^WT^ and Vav1^GEF−^ T cells generated by Vav^NULL^-HSCC were stimulated with anti-CD3 antibodies, in the presence or absence of anti-CD28 antibodies, and proliferation was measured by ^3^H-thymidine incorporation (Fig. 5A). While, as we have previously shown, Vav^NULL^ T cells showed essentially no proliferation in this assay [29], surprisingly, Vav1^GEF−^ T cells showed a robust response that was similar to Vav1^WT^ at all concentrations of stimulatory antibodies tested (Fig. 5A and data not shown). As an alternative measure of T cell proliferation, we used CFSE dye-dilution assays, which also showed comparable proliferative responses of Vav1^GEF−^ and Vav1^WT^ T cells (Fig. 5B). Moreover, analyses of T cell proliferation induced by superantigen SEE-pulsed APCs showed similar results (Fig. 5C,D), indicating that the intrinsic Vav GEF activity is not required for T cell proliferation.

Since Vav1-deficiency has been shown to impair generation of effector T cells and cytokine production with deficient IL-4 expression and enhanced Th1 development [41], we examined if Vav1 GEF activity may be essential in this process. To this end, purified naïve CD4^+^CD62L^hi^ T cells from Vav1^WT^ or Vav1^GEF−^ mice were stimulated under Th1 or Th2 polarizing conditions and then assayed for IFNγ or IL-4 production. Results of these experiments showed similar cytokine production profiles of Vav1^WT^ and Vav1^GEF−^ T cells (Fig. 5E). Taken together, these experiments indicate that while Vav proteins are essential for the induction of T cell proliferative responses, the intrinsic GEF activity appears dispensable for Vav function in T cells. Of note, while previous reports indicated involvement of Vav GEF activity in CD28 signaling (reviewed in [42]), our results suggest that there may also exist a GEF-independent mechanism for Vav-mediated CD28 co-stimulation.

### Expression of Vav1^GEF−^ rescues defects in TCR signaling, actin cytoskeleton remodeling, Rac1 activation, and MTOC polarization

**Figure 6:**
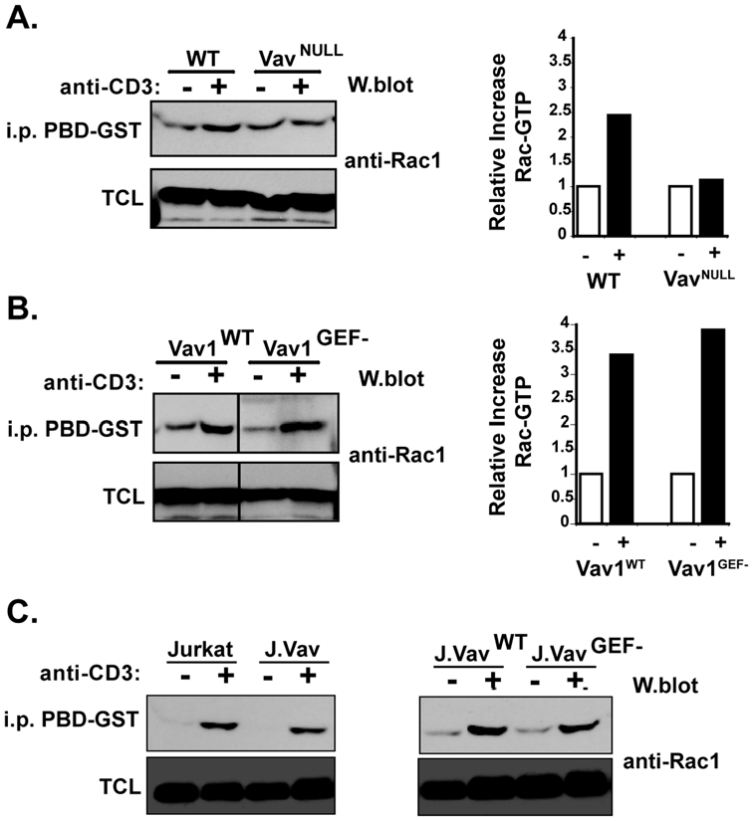
Expession of Vav1^GEF−^ restores TCR-induced Rac1 activation. (A) Rac1 activation in WT and Vav^NULL^ T cells stimulated with anti-CD3 antibodies. The graph represents relative increase in Rac1-GTP. (B) Rac1 activation in Vav1^WT^ and Vav1^GEF−^ T cells stimulated with anti-CD3 antibodies, n = 2. The graph represents relative increase in Rac1-GTP. (C) Rac1 activation in Jurkat, J.Vav, J.Vav reconstituted with WT Vav1 (J.Vav^WT^), and J.Vav reconstituted with GEF-deficient Vav1 (J.Vav^GEF−^). TCL = total cell lysate.

Our previous studies showed defects in TCR-induced Ca^++^ and Ras/MAPK signaling in Vav^NULL^ T cells [29], however it is not known if the intrinsic Vav GEF activity is required in these processes. To address this issue, we examined Ca^++^ mobilization in response to TCR stimulation in Vav1^WT^ and Vav1^GEF−^ T cells and found that both types of cells showed a similar response (Fig. 5F). Similarly, activation of Erk-1/2 appeared normal in both Vav1^WT^ and Vav1^GEF−^ T cells (Fig. 5G). These results indicate that although the activation of Ca^++^ and Erk signaling downstream of the TCR requires Vav [29], it does not depend on the intrinsic Vav GEF activity. In this context, in accord with reports of a defect in TCR activation of Rac1 in Vav1^−/−^ T cells, [43], [44], we also found defective TCR-induced Rac1 activation in Vav^NULL^ T cells and a modest reduction in Rac1 activation in J.Vav cells (Fig. 6). Given the disruption of Ca^++^ and MAPK signaling in Vav^NULL^ T cells, we reasoned that defective Rac activation in these cells likely results from the loss of Vav linker function. Consistent with this view, the induction of activated Rac1 in TCR-stimulated Vav1^WT^ and Vav1^GEF-^ T cells was similar, as was that in J.Vav^WT^ compared to J.Vav^GEF−^ (Fig. 6), indicating that Vav GEF activity is dispensable for TCR induction of Rac1.

**Figure 7:**
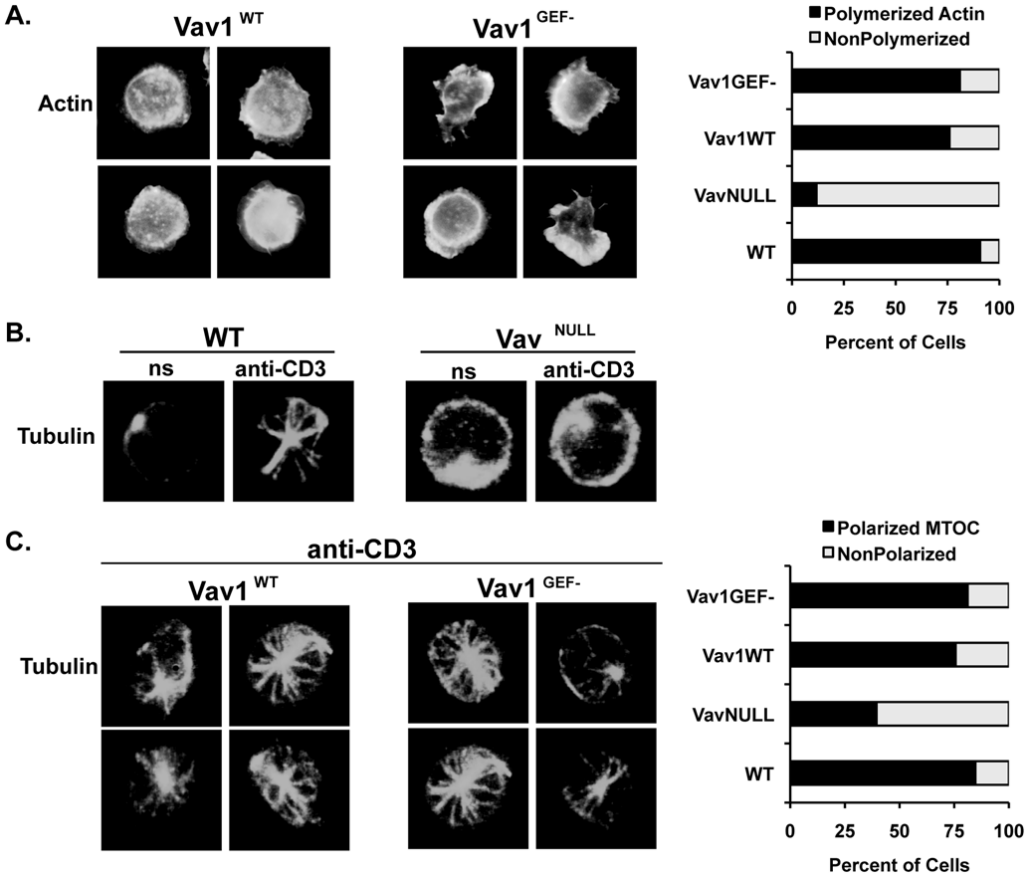
Expression of Vav1^GEF−^ restores TCR-induced actin polymerization and MTOC polarization in Vav^NULL^ T cells. (A) Left panels: Actin polymerization of purified Vav1^WT^ and Vav1^GEF−^ T cells incubated on anti-CD3 antibody-coated coverslips as described in Figure 1. Right panel: Graphical display of the percentages of the indicated T cells that exhibited actin polymerization after stimulation, n>400 cells per group. (B) Purified T cells from WT and Vav^NULL^ mice were either incubated on PBS-coated coverslips (ns = nonstimulated) or were incubated on anti-CD3 antibody-coated coverslips for 30 minutes and MTOCs visualized by staining with fluorescein (FITC)-anti-α-tubulin. (C) Left panels: Purified T cells from Vav1^WT^ and Vav1^GEF−^ mice were incubated on anti-CD3 antibody-coated coverslips for 30 minutes and MTOCs visualized as described in (B). Right panel: Graphical display of the percentages of the indicated T cells that exhibited MTOC polarization after stimulation, n>152 cells per group.

To examine if the GEF activity of Vav is essential for TCR-induced actin polymerization, Vav1^WT^ or Vav1^GEF−^ T cells were incubated on stimulatory coverslips, and F-actin structures were visualized as in Fig. 1. While Vav^NULL^ T cells completely failed to spread and form lamellipodia or filopodia following TCR stimulation (Fig. 1, Table 1), both Vav1^WT^ and Vav1^GEF−^ T cells showed robust actin polymerization and spreading, virtually indistinguishable from that of WT T cells (Fig. 7A, Table 1). These data indicate that while Vav proteins are indispensable for TCR-induced F-actin remodeling (Fig. 1), the intrinsic GEF activity does not appear to be required in this process.

**Table 1:** TCR-induced F-actin polymerization in T cells activated on stimulatory coverslips.

	Total Cells Counted	# Actin Polymerized	% Actin Polymerized
WT	443	402	90.7%
Vav^NULL^	427	51	11.9%
Vav1^WT^	434	330	76.0%
Vav1^GEF−^	400	324	81.0%

Since a recent report implicated Vav in TCR-induced MTOC polarization [26], we examined the requirement for Vav GEF activity in this process. These experiments showed that while MTOC polarization in Vav^NULL^ T cells was reduced essentially to background levels, as compared to WT (Fig. 7B, Table 2), MTOC polarization in Vav1^WT^ and Vav1^GEF−^ cells was similar to WT (Fig. 7C, Table 2). Collectively, these data indicate that while T cells require Vav proteins for TCR signaling and cytoskeletal regulation, Vav GEF activity appears dispensable. Therefore, Vav appears to mediate TCR signals as a critical linker protein rather than as a *bona fide* Rho GEF.

**Table 2:** TCR-induced MTOC polarization in T cells activated on stimulatory coverslips.

	Total Cells Counted	# MTOC Polarized	% MTOC Polarized
WT	197	167	84.7%
Vav^NULL^	195	77	39.5%
Vav1^WT^	152	115	75.7%
Vav1^GEF−^	165	134	81.2%

## Discussion

Stimulation of T cells with anti-CD3 antibodies immobilized on a planar surface permits analyses of the initial formation and the stability of TCR-induced signaling microclusters, or proto-synapses, in live cells [3], [4], [5], [6], [7], [33]. In this regard, following contact of a T cell with a stimulatory surface, ZAP-70, SLP-76, LAT, GADS, and Grb2 are quickly incorporated into signaling microclusters [4], [5], [33]. Here, we report that Vav1 rapidly assembles into TCR-induced microclusters, and remains stable and lacks lateral motion. A recent adaptation to visualizing the dynamic redistribution of TCR-induced microclusters involves stimulation of T cells with cognate TCR ligands embedded in fluid lipid bilayers instead of immobilized anti-CD3. Although this approach allows engaged TCRs to diffuse freely throughout the T cell membrane and to coalesce at the cSMAC within the immunological synapse [7], [8], unlike immobilized anti-CD3 stimulation, data generated using either approach indicates that signaling microclusters form at early time points following TCR stimulation and are relevant sites of TCR signaling initiation and maintenance.

Vav1 may interact with the TCR/CD3-complex in several different ways, including via direct interaction with TCRζ chains [45] or by binding to ZAP-70 or SLP-76 [46], [47]. In this regard, together with the observation that Vav1 is rapidly recruited to signaling microclusters at the initial sites of actin polymerization, similar to other essential linkers such as LAT, these results indicate that Vav itself may function as a linker in TCR-induced actin polymerization, independently of its other potential role as a Rho GTPase activator. Thus, given that Vav recruitment to ZAP-70, SLP-76, or LAT is dependent upon tyrosine phosphorylation [47], [48], our results support a model in which TCR-induced actin polymerization is initiated in the context of phosphorylated ITAMs. Consistent with this view, our analyses indicate that Vav colocalizes with other linkers, such as SLP-76, in TCR-induced microclusters that rapidly form at TCR contacts.

Although Vav1-deficient T lymphocytes and J.Vav cells show defects in TCR signaling, surprisingly little or no evidence exists in support of the requirement for Vav1 for TCR-induced actin polymerization. In this regard, two main issues appear to have precluded significant inroads. First, the functional redundancy of Vav proteins, all of which are expressed in T cells, produces compensatory effects in cells lacking individual family members. Second, in studies with T cell-APC conjugates or with other systems involving an immune synapse, Vav-dependent signals emanating from integrins and/or costimulatory molecules are difficult to discriminate from the TCR-specific signals that may depend upon Vav. Therefore, in this report we examined the requirement for the entire Vav family in actin reorganization using Vav^NULL^ T cells and anti-CD3 stimulation on a planar surface and find a virtually complete disruption in actin polymerization, which is the first such direct demonstration. Strikingly, these defects are rescued by expression of GEF-inactive Vav. In this regard, several potential scenarios could explain the lack of requirement for the intrinsic Vav GEF activity. For example, a previously described SLP-76-Nck-WASp complex may control actin reorganization independent of Rho-protein involvement [6], [15], [16]. Alternatively, a recently described Dynamin2 function in TCR-induced actin polymerization could contribute Vav-dependent, but GEF activity-independent, effects [49].

While the requirement for Vav SH2, SH3, CH, and PH domains for Vav function in TCR signaling is well established [43], [47], [50], [51], the requirement for the GEF activity remains controversial [30], [34], [50], [52], [53]. In this regard, the truncated form of Vav1 (with constitutive GEF activity) does not enhance NFAT-dependent transcription [30], [47], suggesting that Vav GEF activity is not sufficient to propagate signals leading to NFAT. However, while several reports indicated that GEF activity of Vav1 may be required in TCR-induced NFAT- and NFκB-mediated transcriptional activation [34], [50], [52], [53], another study showed that Vav1 GEF activity is not required in enhancing NFAT-dependent transcription [30]. Several potential explanations exist for these apparent discrepancies. For example, the effects of Vav1 may vary depending on relative levels of protein expression, as transient expression of Vav1 in Jurkat cells was shown to potently stimulate NFAT-dependent signaling, even in the absence of TCR engagement [30], [47]. In this context, overexpression of GEF-inactive Vav1 could conceivably result in dominant negative effects on gene transcription [53]. Alternatively, ectopic expression of GEF-inactive Vav1 could exert positive effects on downstream signaling pathways, for example via a mechanism involving *trans*-complementation of the missing GEF activity by the activity of endogenous (GEF-sufficient) Vav1 protein [30]. Here, we show that GEF-inactive Vav1, expressed stably at endogenous levels in J.Vav cells, rescues TCR-induced NFAT- and NFκB-dependent transcription. In this regard, we used a previously characterized L278Q loss-of-function mutant [22], [35] and verified the loss of catalytic activity by comprehensive analyses of GDP/GTP exchange *in vitro* and by *in vivo* assays for F-actin induction by the N-terminally truncated Vav (Fig. S4 and [9], [36]). However, these experiments do not rule out the possibility that the GEF activity of other Vav proteins, Vav2 and/or Vav3, both of which are expressed in J.Vav cells, may contribute compensatory effects to Vav1^GEF−^-mediated signals. To address this issue, we used Vav^NULL^ T cells that lack all three endogenous Vav proteins. We note, however, that other non-Vav GEFs could also be responsible for contributing compensatory activity, such as βPIX, which is activated in response to TCR stimulation in J.Vav cells [54].

While mice lacking individual Vav family proteins show partial to no defects in T lymphocytes, Vav^NULL^ mice show a severe block in T cell development [29], [38], [39], [40], [55]. We reasoned, given our earlier observation that Vav1^+/−^/Vav2^−/−^/Vav3^−/−^ mice (which express only Vav1 but not Vav2 or Vav3) show no discernible defects in T-lineage cells [29], that reintroduction of Vav1 alone should be sufficient to rescue Vav^NULL^ T cell development and function. Indeed, we found that the expression of Vav1^WT^ is capable of completely restoring development and activation of Vav^NULL^ T cells. The levels of expression of recombinant Vav1 in these “rescued” T cells closely approximate that of endogenous Vav1 in WT T cells (Fig. 4C), a finding that is notable because in T cells generated by the Vav^NULL^-HSCC assay, Vav1 expression is not controlled by the endogenous promoter elements but rather by retroviral-based LTRs. Thus, these data indicate that one or more mechanisms may regulate Vav1 expression in T cells, or possibly, this could be due to a developmental advantage of T cell progenitors that express a certain level of Vav1. While at present we do not completely understand how the levels of Vav1 expression may be regulated in T cells, expression of either WT or GEF-inactive Vav1, at levels indistinguishable from endogenous Vav1, can support T cell development.

Consistent with recent studies implicating Vav1 in control of microtubular reorganization [26], Vav^NULL^ T cells show disrupted MTOC polarization (Fig. 7, Table 2). While this function of Vav could, conceivably, require the GEF activity for activation of GTPases such as Cdc42 that can modulate MTOC polarization [56], analyses of Vav1^GEF−^ T cells suggest that Vav effects on MTOC polarization are Vav GEF-independent. However, while Vav appears to be essential for both TCR-mediated regulation of MTOC polarization and actin polymerization, any GEF activity(ies) required in these processes could be controlled by other effectors such as αPIX, βPIX, DOCK2, or DOCK180, or the RhoA effectors p160ROCK and p190RhoGEF [19], [20], [21], [23], [24], [57]. However, recent studies clearly show that WASp/WAVE-mediated actin polymerization can be induced by the Arp2/3 complex independently of Rho GTPases, for example via binding of Nck [3], [6]. In this context, the rescue of TCR-induced F-actin defects in Vav^NULL^ T cells by GEF-inactive Vav expression indicates that the intrinsic Vav GEF activity is not essential for actin polymerization downstream of the TCR. These data suggest that Vav functions as a TCR-proximal linker critical for cytoskeletal reorganization that could be Rho GTPase-independent. Interestingly, similar to Vav, the Rac-GEF kalirin induces lamellipodia formation independently of its intrinsic GEF activity [58] suggesting that regulation of actin dynamics by some GEFs may not require the catalytic activity of the DH domain.

Alternatively, however, Rac activation downstream of the TCR may be mediated by other Rho-GEFs, such as αPIX, βPIX, or DOCK2. Indeed, T cells deficient in DOCK2 show defective Rac1 activation by the TCR, but unlike Vav^NULL^ T cells, show no defects in Ca^++^ or MAPK signaling [24], indicating distinct mechanisms for regulation of Rac and Ras GTPases downstream of the TCR. In this regard, the function of Vav appears to be as a TCR linker required for both Rac and Ras signaling. Thus, taken together, the reduction of specific catalytic activity of the Vav GEF-mutant used in our study to essentially undetectable levels (less than 1% of wild type), combined with no evidence for any local increases in the concentration of the mutant protein, as judged by TIRFM analyses of activated T cells, and no evidence of any titratable differences in the ability of Vav1^GEF−^ T cells to respond to TCR stimulation, provide compelling evidence that defects in TCR signaling (including Ca^++^, MAPK, and Rac1 activation), actin polymerization, MTOC polarization and proliferation of Vav^NULL^ T cells are due to the loss of adaptor/linker, rather than GEF, function of Vav. Consistent with effective reduction of the GEF activity of the Vav1^GEF−^ constructs, combining the GEF-killing mutation with the GEF-activating mutation (Y174F) completely abolished the effects of the latter [9]. Of note, we obtained similar results using another GEF-inactive form of Vav (Vav1^E201A/K335A^) [59], [60], [61] (data not shown). Thus, the preponderance of evidence presented in our report indicates to us that a scenario in which any residual GEF activity would account for the rescue of T cell function by the Vav1 L278Q mutant is unlikely. Moreover, recent reports demonstrate that expression of the same Vav1^GEF−^ mutant (L278Q) in Vav-deficient myeloid cells does not rescue LPS- or FcγR-triggered oxidative burst [36], [37], indicating that in these cells the intrinsic GEF activity of Vav is essential for its function, in contrast to TCR-induced signaling.

We also note that because mice congenitally lacking Vav1 show primarily T-lineage specific defects [62], one could reason that the intrinsic GEF-activity of Vav1 could be an attractive potential target for pharmacological inhibition in the context of T cell-directed immunosuppressive therapies. However, our data presented here suggest that the inhibition of the Vav1 enzymatic activity as a GEF would likely not be an effective strategy for suppressing T cell activation and proliferative expansion.

While Vav proteins also contain a PH domain, implicated in PIP_2_ and PIP_3_ binding and the regulation of Vav plasma membrane interactions as well as GEF activity [25], a recent study showed that a mutation rendering Vav1 PH domain incapable of binding to phosphatidylinositol metabolites leads to TCR signaling defects [43]. Thus, given the results of our studies presented in this report, it is possible that the PH domain could contribute to Vav function in TCR signaling independently of its effects on GEF activity.

Vav has been among the first phosphotyrosine-proteins identified in TCR signaling pathways [63], [64] and indeed, tyrosine phosphorylation distinguishes the Vav family from a plethora of other Dbl proteins. While Vav tyrosine phosphorylation has mainly been considered in the context of the regulation of the intrinsic GEF activity [31], [65], our data presented in this report suggest that tyrosine phosphorylation of Vav could also contribute to its function as a TCR linker for activated T cells. In this regard, we propose that Vav mediates TCR signals in a GEF-independent manner.

## Materials and Methods

### Generation of Reconstituted J.Vav Cell Lines, Stimulation, and Immunoblotting

The Vav1-deficient J.Vav cell line was previously described [34]. To generate J.Vav cell lines expressing Vav1^WT^ or Vav1^GEF−^ proteins, GFP-tagged Vav1 expression constructs were transduced into J.Vav cells via “spinfection” with retroviral particles at RT, 2000 rpm for 90 mins. GFP^+^ cells were FACS sorted and subcloned. Vav1-GFP constructs were generated by ligation of an XbaI-BamHI Vav1-GFP cDNA fragment into IRES-GFP-RV digested with XhoI-BamHI replacing IRES-GFP. Mutagenesis was performed by PCR (Quickchange kit, Stratagene, La Jolla, CA) and confirmed by sequencing. Cells were stimulated with anti-CD3ε (clone HIT3a; 1 µg/mL, BD Biosciences, San Diego, CA)+anti-IgG2a (1 µg/mL, Southern Biotechnology Assoc., Birmingham, AL), as indicated, and lysed in RIPA buffer supplemented with a protease inhibitor cocktail (Boehringer, Ridgefield, CT), 10 mM NaF, and 1 mM Na_3_VO_4_. Western blotting was performed following standard procedures. Primary antibodies were developed with HRP-conjugated secondary antibodies (anti-mouse, Zymed, San Francisco, CA; anti-rabbit, Amersham Biosciences, Piscataway, NJ; anti-sheep, Upstate, Lake Placid, NY). Immune complexes were detected by enhanced chemiluminescence (Amersham Biosciences).

*TIRFM Imaging*. Imaging of dynamic Vav1-GFP microcluster assembly and movement was performed using TIRF microscopy as described in [9], [66]. Image recording and processing were performed using AQUACOSMOS software (Hamamatsu Photonics, Japan) and image analyses were performed using Metamorph Software (Molecular Devices Corp., Sunnyvale, CA). Kymographic analysis was performed as in [9]. See Supplemental Methods S1 for more extensive descriptions.

### Actin Polymerization and MTOC Polarization

T cells were purified from LN cell suspensions by removal of B cells with anti-Ig-coated Dynabeads (Invitrogen, Carlsbad, CA) using standard procedures. T cells were resuspended in plain DMEM and incubated on anti-CD3ε-coated coverslips (clone 145-2C11, 1 µg/mL, BD Biosciences) for the indicated time points. Actin polymerization was visualized by staining of F-actin with Alexa-Fluor-488-phalloidin (Molecular Probes, Eugene, OR). MTOC polarization was performed as previously described [67]. MTOCs were visualized by staining with fluorescein (FITC)-anti-α-tubulin (Sigma, St. Louis, MO). Confocal and differential interference contrast (DIC) images were taken using Zeiss LSM510 confocal system and analyzed by ImageJ software and LSM Image Browser software.

### Luciferase Assays

Cells were transfected with 5 µg luciferase plasmid containing NFATx3 binding sites from the IL-2 promoter, or NFκBx2 binding sites from the IFNβ promoter. Sixteen hours following transfection, cells were either left unstimulated or stimulated with anti-CD3+anti-IgG2a for 6 hours. Luciferase assays were then performed according to manufacturer's instructions (Promega, Madison, WI).

### Mice, Cell Suspensions, Antibodies, and Flow Cytometry

Germline Vav1^−/−^ and Vav^NULL^ mice have been previously described [29], [55] and were maintained in the SPF facility of Washington University School of Medicine according to institutional protocols. Cell suspensions were prepared, counted, and stained with antibodies following standard procedures. The following antibody conjugates were used (BD Biosciences): phycoerythrin (PE) and allophyocyanin (APC)-H129.19 (anti-CD4) and cytochrome C (CyC)-53–6.7 (anti-CD8α). All samples were analyzed on a FACSCalibur flow cytometer (Becton Dickinson) with FlowJo software.

### Vav^NULL^ hematopoietic stem cell complementation (Vav^NULL^-HSCC)

A single dose of 150 mg/kg of 5-flurouracil (10 mg/mL in PBS, Sigma) was injected into donor mice intraperitoneally. Four to five days post-injection, donors were sacrificed, and bone marrow (BM) harvested. BM cells were expanded in media containing 15% FCS and supplemented with SCF (100 ng/ml, PeproTech, Rocky Hill, NJ), IL-3 (6 ng/ml, PeproTech), and IL-6 (10 ng/ml, PeproTech). After 2 days in culture, the cells were retrovirally transduced via “spinfection.” Infection efficiency and viability of BM cells was assessed by flow cytometry. RAG2^−/−^ recipient mice were lethally irradiated with 950 Rad (gamma irradiation (Cs^137^), MDS Nordion, Ottawa, Ontario, Canada) and injected with 250 µL cell suspension (∼.25×10^6^ cells) invtravenously. Chimera were sacrificed and analyzed 5–7 weeks following reconstitution.

### T Cell Stimulation and Proliferation Assays

Purified T cells were stimulated with soluble anti-CD3ε antibodies (clone 145-2C11, 1 µg/mL, BD Biosciences)+/− anti-CD28 (clone 37.51, .5 µg/mL, BD Biosciences), or SEE (1 ng/mL, Toxin Technologies, Sarasota, FL) as indicated, and ^3^H-thymidine incorporation performed as described in [29]. For CFSE labeling, cells were labeled with 1 µM CFSE (Molecular Probes) and stimulated, as indicated, for 72 hrs. Cells were stained with anti-CD4-APC conjugates and proliferation analyzed by flow cytometry.

### T Cell Polarization and Analysis of Cytokine Production

Naïve CD4^+^CD62L^+^ LN T cells FACS sorted from fresh LN were activated and polarized to Th1 or Th2 as previously described in [68]. For ELISA, resting cells were stimulated with anti-CD3 antibodies for 24 hrs. Cytokine concentrations were measured in culture supernatants using Cytometric Bead Array (BD Biosciences) according to manufacturer's instructions.

### Ca^++^ Fluxes and MAPK Activation

Ca^++^ signaling was measured by loading total LN cell suspensions with Fluo-4-AM (3–5 µg/mL, Molecular Probes). Cells were stained with anti-CD4-APC conjugates and analyzed by flow cytometry as described in [29]. Erk1/2 signaling was measured as previously described [29].

*Rac assay*. Purified LN T cells were starved for 30 mins in media lacking serum. Cells were treated with 1 µg/mL anti-CD3 antibodies for 2 mins and Rac assay performed using EZ-Detect Rac1 Activation Kit (Pierce, Rockford, IL) according to manufacture's instructions.

### Purification of GST-Rac1 and MBP-Vav1, and guanine nucleotide exchange assays

Bacterially expressed GST-Rac1 was purified as previously described in [69]. MBP-Vav1 fusion proteins were expressed in E. coli strain BL21(DE) followed by purification using amylose resin according to the manufacture's protocol (NEB, Beverley, MA), with the exception that the column was washed with 20 mM Tris, pH 7.4, 200 mM NaCl after binding the protein to the resin. The MBP-fusion proteins were eluted with the same buffer containing 10 mM maltose. Exchange assays were performed essentially as described in [70], [71].

### Statistical Analysis

Data are expressed throughout as mean+standard deviation. Data sets derived from the indicated genotypes were compared using the two-tailed unpaired Student's t-test. Differences were considered statistically significant when p<0.05.

## Supporting Information

Figure S1 Vav1-deficient T cells show minimal defects in TCR-induced actin cytoskeletal reorganization and cell spreading. (A) Purified LN T cells from WT or Vav1−/− mice were plated on coverslips coated with anti-CD3 antibodies, and cells were subsequently stained for F-actin. Images captured by confocal microscopy depict the cell membrane-coverslip interface in the XY plane as well as Z-stacked images of the entire cell. Images shown are representative of n>10 cells for each stimulation condition. (B) T cells were stimulated and stained as in (A). Cell spreading was determined by measuring the perimeter of the membrane-coverslip interface as defined by F-actin staining. Measurements were made for n>10 cells per stimulation condition. (C) T cells from (B) were analyzed for F-actin content at the membrane-coverslip interface by measuring the pixel intensity of Alexa-Fluor-488-phalloidin fluorescence within the area defined by the perimeter of the membrane-coverslip interface (integrated density). Measurements were performed in n>10 cells per condition.
(0.33 MB TIF)

Figure S2 Vav1 microcluster formation is induced by TCR stimulation. Live J.Vav cells expressing GFP-only, or J.Vav1WT cells were incubated on coverslips coated with anti-CD3 antibodies, or with poly L-lysine and imaged in real time using TIRFM.
(0.34 MB TIF)

Figure S3 Vav1 colocalizes with SLP-76 in TCR-induced microclusters. J.Vav, J.Vav1WT, or J.Vav1GEF- cells were activated on anti-CD3-coated coverslips for 2 minutes followed by fixation and permeabilization. SLP-76 microclusters were visualized by staining with anti-SLP-76 antibodies followed by anti-rabbit-Cy5. Vav1 microclusters are GFP+. Images were captured by confocal imaging of cells within the plane of contact with the stimulatory coverslip, shown by internal reflection microscopy (IRM). Representative images are shown (n≥10).
(2.27 MB TIF)

Figure S4 The Vav1 L278Q mutation abrogates GEF activity. (A) Stable expression of GFP-tagged Vav1WT and Vav1GEF- in J.Vav cells was similar to endogenous levels of Vav1 in Jurkats as demonstrated by immunoblotting with anti-Vav1 antibodies and by FACS. (B) (left) In vitro GDP-GTP exchange on increasing concentrations of Rac1 was measured as loss of radiolabeled [3H]-GDP in the presence of unlabeled GTP and a WT Vav1 MBP-DH-PH-ZF fusion protein or (middle) a fusion protein containing the Vav1 DH domain expressing L278Q (MBP-DH(L278Q)-PH-ZF), corresponding to L213Q in N-terminally truncated “onco” Vav, [22], [35]. (right) Kinetics of in vitro GDP-GTP exchange as shown in left and middle panels. Bottom panel: kinetic values for GDP-GTP exchange on Rac1 by WT Vav1 MBP-DH-PH-ZF or GEF-inactive MBP-DH(L278Q)-PH-ZF, as determined by Lineweaver-Burk plot shown above.
(0.17 MB TIF)

Figure S5 Activation of NFAT and NFkB luciferase by PMA and Ionomycin is Vav-independent. NFAT (A) or NFκB (B) luciferase reporter assays of untreated and PMA and ionomycin-activated J.Vav, J.Vav1WT and J.Vav1GEF- cells. Data are mean±SD n>5 experiments.
(0.09 MB TIF)

Supplemental Methods S1 (0.06 MB DOC)

## References

[1] Smith-Garvin JE, Koretzky GA, Jordan MS (2009). T cell activation.. Annu Rev Immunol.

[2] Kane LP, Lin J, Weiss A (2000). Signal transduction by the TCR for antigen.. Curr Opin Immunol.

[3] Bunnell SC, Kapoor V, Trible RP, Zhang W, Samelson LE (2001). Dynamic actin polymerization drives T cell receptor-induced spreading: a role for the signal transduction adaptor LAT.. Immunity.

[4] Bunnell SC, Hong DI, Kardon JR, Yamazaki T, McGlade CJ (2002). T cell receptor ligation induces the formation of dynamically regulated signaling assemblies.. J Cell Biol.

[5] Bunnell SC, Singer AL, Hong DI, Jacque BH, Jordan MS (2006). Persistence of cooperatively stabilized signaling clusters drives T-cell activation.. Mol Cell Biol.

[6] Barda-Saad M, Braiman A, Titerence R, Bunnell SC, Barr VA (2005). Dynamic molecular interactions linking the T cell antigen receptor to the actin cytoskeleton.. Nat Immunol.

[7] Yokosuka T, Sakata-Sogawa K, Kobayashi W, Hiroshima M, Hashimoto-Tane A (2005). Newly generated T cell receptor microclusters initiate and sustain T cell activation by recruitment of Zap70 and SLP-76.. Nat Immunol.

[8] Campi G, Varma R, Dustin ML (2005). Actin and agonist MHC-peptide complex-dependent T cell receptor microclusters as scaffolds for signaling.. J Exp Med.

[9] Miletic AV, Sakata-Sogawa K, Hiroshima M, Hamann MJ, Gomez TS (2006). Vav1 acidic region tyrosine 174 is required for the formation of T cell receptor-induced microclusters and is essential in T cell development and activation.. J Biol Chem.

[10] Huang Y, Burkhardt JK (2007). T-cell-receptor-dependent actin regulatory mechanisms.. J Cell Sci.

[11] Fuller CL, Braciale VL, Samelson LE (2003). All roads lead to actin: the intimate relationship between TCR signaling and the cytoskeleton.. Immunol Rev.

[12] Billadeau DD, Nolz JC, Gomez TS (2007). Regulation of T-cell activation by the cytoskeleton.. Nat Rev Immunol.

[13] Gomez-Rodriguez J, Readinger JA, Viorritto IC, Mueller KL, Houghtling RA (2007). Tec kinases, actin, and cell adhesion.. Immunol Rev.

[14] Gil D, Schamel WW, Montoya M, Sanchez-Madrid F, Alarcon B (2002). Recruitment of Nck by CD3 epsilon reveals a ligand-induced conformational change essential for T cell receptor signaling and synapse formation.. Cell.

[15] Sasahara Y, Rachid R, Byrne MJ, de la Fuente MA, Abraham RT (2002). Mechanism of recruitment of WASP to the immunological synapse and of its activation following TCR ligation.. Mol Cell.

[16] Zeng R, Cannon JL, Abraham RT, Way M, Billadeau DD (2003). SLP-76 coordinates Nck-dependent Wiskott-Aldrich syndrome protein recruitment with Vav-1/Cdc42-dependent Wiskott-Aldrich syndrome protein activation at the T cell-APC contact site.. J Immunol.

[17] Badour K, Zhang J, Shi F, Leng Y, Collins M (2004). Fyn and PTP-PEST-mediated regulation of Wiskott-Aldrich syndrome protein (WASp) tyrosine phosphorylation is required for coupling T cell antigen receptor engagement to WASp effector function and T cell activation.. J Exp Med.

[18] Rohatgi R, Nollau P, Ho HY, Kirschner MW, Mayer BJ (2001). Nck and phosphatidylinositol 4,5-bisphosphate synergistically activate actin polymerization through the N-WASP-Arp2/3 pathway.. J Biol Chem.

[19] Feng Q, Albeck JG, Cerione RA, Yang W (2002). Regulation of the Cool/Pix proteins: key binding partners of the Cdc42/Rac targets, the p21-activated kinases.. J Biol Chem.

[20] Bagrodia S, Taylor SJ, Jordon KA, Van Aelst L, Cerione RA (1998). A novel regulator of p21-activated kinases.. J Biol Chem.

[21] Yoshii S, Tanaka M, Otsuki Y, Wang DY, Guo RJ (1999). alphaPIX nucleotide exchange factor is activated by interaction with phosphatidylinositol 3-kinase.. Oncogene.

[22] Crespo P, Bustelo XR, Aaronson DS, Coso OA, Lopez-Barahona M (1996). Rac-1 dependent stimulation of the JNK/SAPK signaling pathway by Vav.. Oncogene.

[23] Manser E, Leung T, Lim L (1998). Identification and characterization of small GTPase-associated kinases.. Methods Mol Biol.

[24] Sanui T, Inayoshi A, Noda M, Iwata E, Oike M (2003). DOCK2 is essential for antigen-induced translocation of TCR and lipid rafts, but not PKC-theta and LFA-1, in T cells.. Immunity.

[25] Turner M, Billadeau DD (2002). VAV proteins as signal integrators for multi-subunit immune-recognition receptors.. Nat Rev Immunol.

[26] Ardouin L, Bracke M, Mathiot A, Pagakis SN, Norton T (2003). Vav1 transduces TCR signals required for LFA-1 function and cell polarization at the immunological synapse.. Eur J Immunol.

[27] Krawczyk C, Oliveira-dos-Santos A, Sasaki T, Griffiths E, Ohashi PS (2002). Vav1 controls integrin clustering and MHC/peptide-specific cell adhesion to antigen-presenting cells.. Immunity.

[28] Faure S, Salazar-Fontana LI, Semichon M, Tybulewicz VL, Bismuth G (2004). ERM proteins regulate cytoskeleton relaxation promoting T cell-APC conjugation.. Nat Immunol.

[29] Fujikawa K, Miletic AV, Alt FW, Faccio R, Brown T (2003). Vav1/2/3-null mice define an essential role for Vav family proteins in lymphocyte development and activation but a differential requirement in MAPK signaling in T and B cells.. J Exp Med.

[30] Kuhne MR, Ku G, Weiss A (2000). A guanine nucleotide exchange factor-independent function of Vav1 in transcriptional activation.. J Biol Chem.

[31] Lopez-Lago M, Lee H, Cruz C, Movilla N, Bustelo XR (2000). Tyrosine phosphorylation mediates both activation and downmodulation of the biological activity of Vav.. Mol Cell Biol.

[32] Amarasinghe GK, Rosen MK (2005). Acidic region tyrosines provide access points for allosteric activation of the autoinhibited Vav1 Dbl homology domain.. Biochemistry.

[33] Houtman JC, Yamaguchi H, Barda-Saad M, Braiman A, Bowden B (2006). Oligomerization of signaling complexes by the multipoint binding of GRB2 to both LAT and SOS1.. Nat Struct Mol Biol.

[34] Cao Y, Janssen EM, Duncan AW, Altman A, Billadeau DD (2002). Pleiotropic defects in TCR signaling in a Vav-1-null Jurkat T-cell line.. EMBO J.

[35] Crespo P, Schuebel KE, Ostrom AA, Gutkind JS, Bustelo XR (1997). Phosphotyrosine-dependent activation of Rac-1 GDP/GTP exchange by the vav proto-oncogene product.. Nature.

[36] Miletic AV, Graham DB, Montgrain V, Fujikawa K, Kloeppel T (2007). Vav proteins control MyD88-dependent oxidative burst.. Blood.

[37] Utomo A, Cullere X, Glogauer M, Swat W, Mayadas TN (2006). Vav proteins in neutrophils are required for FcgammaR-mediated signaling to Rac GTPases and nicotinamide adenine dinucleotide phosphate oxidase component p40(phox).. J Immunol.

[38] Tarakhovsky A, Turner M, Schaal S, Mee PJ, Duddy LP (1995). Defective antigen receptor-mediated proliferation of B and T cells in the absence of Vav.. Nature.

[39] Fischer KD, Zmuldzinas A, Gardner S, Barbacid M, Bernstein A (1995). Defective T-cell receptor signalling and positive selection of Vav-deficient CD4+ CD8+ thymocytes.. Nature.

[40] Zhang R, Alt FW, Davidson L, Orkin SH, Swat W (1995). Defective signalling through the T- and B-cell antigen receptors in lymphoid cells lacking the vav proto-oncogene.. Nature.

[41] Tanaka Y, So T, Lebedeva S, Croft M, Altman A (2005). Impaired IL-4 and c-Maf expression and enhanced Th1-cell development in Vav1-deficient mice.. Blood.

[42] Acuto O, Michel F (2003). CD28-mediated co-stimulation: a quantitative support for TCR signalling.. Nat Rev Immunol.

[43] Prisco A, Vanes L, Ruf S, Trigueros C, Tybulewicz VL (2005). Lineage-specific requirement for the PH domain of Vav1 in the activation of CD4+ but not CD8+ T cells.. Immunity.

[44] Reynolds LF, Smyth LA, Norton T, Freshney N, Downward J (2002). Vav1 transduces T cell receptor signals to the activation of phospholipase C-gamma1 via phosphoinositide 3-kinase-dependent and -independent pathways.. J Exp Med.

[45] Huang J, Sugie K, La Face DM, Altman A, Grey HM (2000). TCR antagonist peptides induce formation of APC-T cell conjugates and activate a Rac signaling pathway.. Eur J Immunol.

[46] Katzav S, Sutherland M, Packham G, Yi T, Weiss A (1994). The protein tyrosine kinase ZAP-70 can associate with the SH2 domain of proto-Vav.. J Biol Chem.

[47] Wu J, Motto DG, Koretzky GA, Weiss A (1996). Vav and SLP-76 interact and functionally cooperate in IL-2 gene activation.. Immunity.

[48] Fang N, Koretzky GA (1999). SLP-76 and Vav function in separate, but overlapping pathways to augment interleukin-2 promoter activity.. J Biol Chem.

[49] Gomez TS, Hamann MJ, McCarney S, Savoy DN, Lubking CM (2005). Dynamin 2 regulates T cell activation by controlling actin polymerization at the immunological synapse.. Nat Immunol.

[50] Zugaza JL, Lopez-Lago MA, Caloca MJ, Dosil M, Movilla N (2002). Structural determinants for the biological activity of Vav proteins.. J Biol Chem.

[51] Billadeau DD, Mackie SM, Schoon RA, Leibson PJ (2000). Specific subdomains of Vav differentially affect T cell and NK cell activation.. J Immunol.

[52] Hehner SP, Hofmann TG, Dienz O, Droge W, Schmitz ML (2000). Tyrosine-phosphorylated Vav1 as a point of integration for T-cell receptor- and CD28-mediated activation of JNK, p38, and interleukin-2 transcription.. J Biol Chem.

[53] Kaminuma O, Deckert M, Elly C, Liu YC, Altman A (2001). Vav-Rac1-mediated activation of the c-Jun N-terminal kinase/c-Jun/AP-1 pathway plays a major role in stimulation of the distal NFAT site in the interleukin-2 gene promoter.. Mol Cell Biol.

[54] Phee H, Abraham RT, Weiss A (2005). Dynamic recruitment of PAK1 to the immunological synapse is mediated by PIX independently of SLP-76 and Vav1.. Nat Immunol.

[55] Turner M, Mee PJ, Walters AE, Quinn ME, Mellor AL (1997). A requirement for the Rho-family GTP exchange factor Vav in positive and negative selection of thymocytes.. Immunity.

[56] Stowers L, Yelon D, Berg LJ, Chant J (1995). Regulation of the polarization of T cells toward antigen-presenting cells by Ras-related GTPase CDC42.. Proc Natl Acad Sci U S A.

[57] Sancho D, Vicente-Manzanares M, Mittelbrunn M, Montoya MC, Gordon-Alonso M (2002). Regulation of microtubule-organizing center orientation and actomyosin cytoskeleton rearrangement during immune interactions.. Immunol Rev.

[58] Schiller MR, Blangy A, Huang J, Mains RE, Eipper BA (2005). Induction of lamellipodia by Kalirin does not require its guanine nucleotide exchange factor activity.. Exp Cell Res.

[59] Hoffman GR, Cerione RA (2002). Signaling to the Rho GTPases: networking with the DH domain.. FEBS Lett.

[60] Worthylake DK, Rossman KL, Sondek J (2000). Crystal structure of Rac1 in complex with the guanine nucleotide exchange region of Tiam1.. Nature.

[61] Zheng Y (2001). Dbl family guanine nucleotide exchange factors.. Trends Biochem Sci.

[62] Tybulewicz VL (2005). Vav-family proteins in T-cell signalling.. Curr Opin Immunol.

[63] Bustelo XR, Ledbetter JA, Barbacid M (1992). Product of vav proto-oncogene defines a new class of tyrosine protein kinase substrates.. Nature.

[64] Margolis B, Hu P, Katzav S, Li W, Oliver JM (1992). Tyrosine phosphorylation of vav proto-oncogene product containing SH2 domain and transcription factor motifs.. Nature.

[65] Aghazadeh B, Lowry WE, Huang XY, Rosen MK (2000). Structural basis for relief of autoinhibition of the Dbl homology domain of proto-oncogene Vav by tyrosine phosphorylation.. Cell.

[66] Tokunaga M, Kitamura K, Saito K, Iwane AH, Yanagida T (1997). Single molecule imaging of fluorophores and enzymatic reactions achieved by objective-type total internal reflection fluorescence microscopy.. Biochem Biophys Res Commun.

[67] Kuhne MR, Lin J, Yablonski D, Mollenauer MN, Ehrlich LI (2003). Linker for activation of T cells, zeta-associated protein-70, and Src homology 2 domain-containing leukocyte protein-76 are required for TCR-induced microtubule-organizing center polarization.. J Immunol.

[68] Afkarian M, Sedy JR, Yang J, Jacobson NG, Cereb N (2002). T-bet is a STAT1-induced regulator of IL-12R expression in naive CD4+ T cells.. Nat Immunol.

[69] Self AJ, Hall A (1995). Purification of recombinant Rho/Rac/G25K from Escherichia coli.. Methods Enzymol.

[70] Hoshino M, Sone M, Fukata M, Kuroda S, Kaibuchi K (1999). Identification of the stef gene that encodes a novel guanine nucleotide exchange factor specific for Rac1.. J Biol Chem.

[71] Mizuno T, Kaibuchi K, Yamamoto T, Kawamura M, Sakoda T (1991). A stimulatory GDP/GTP exchange protein for smg p21 is active on the post-translationally processed form of c-Ki-ras p21 and rhoA p21.. Proc Natl Acad Sci U S A.

