# Supplementary figures and images for: Vav Links the T Cell Antigen Receptor to the Actin Cytoskeleton and T Cell Activation Independently of Intrinsic Guanine Nucleotide Exchange Activity
Source: PLoS One. 2009 Aug 12;4(8):e6599. doi: 10.1371/journal.pone.0006599 (PMC2719804; doi:10.1371/journal.pone.0006599)

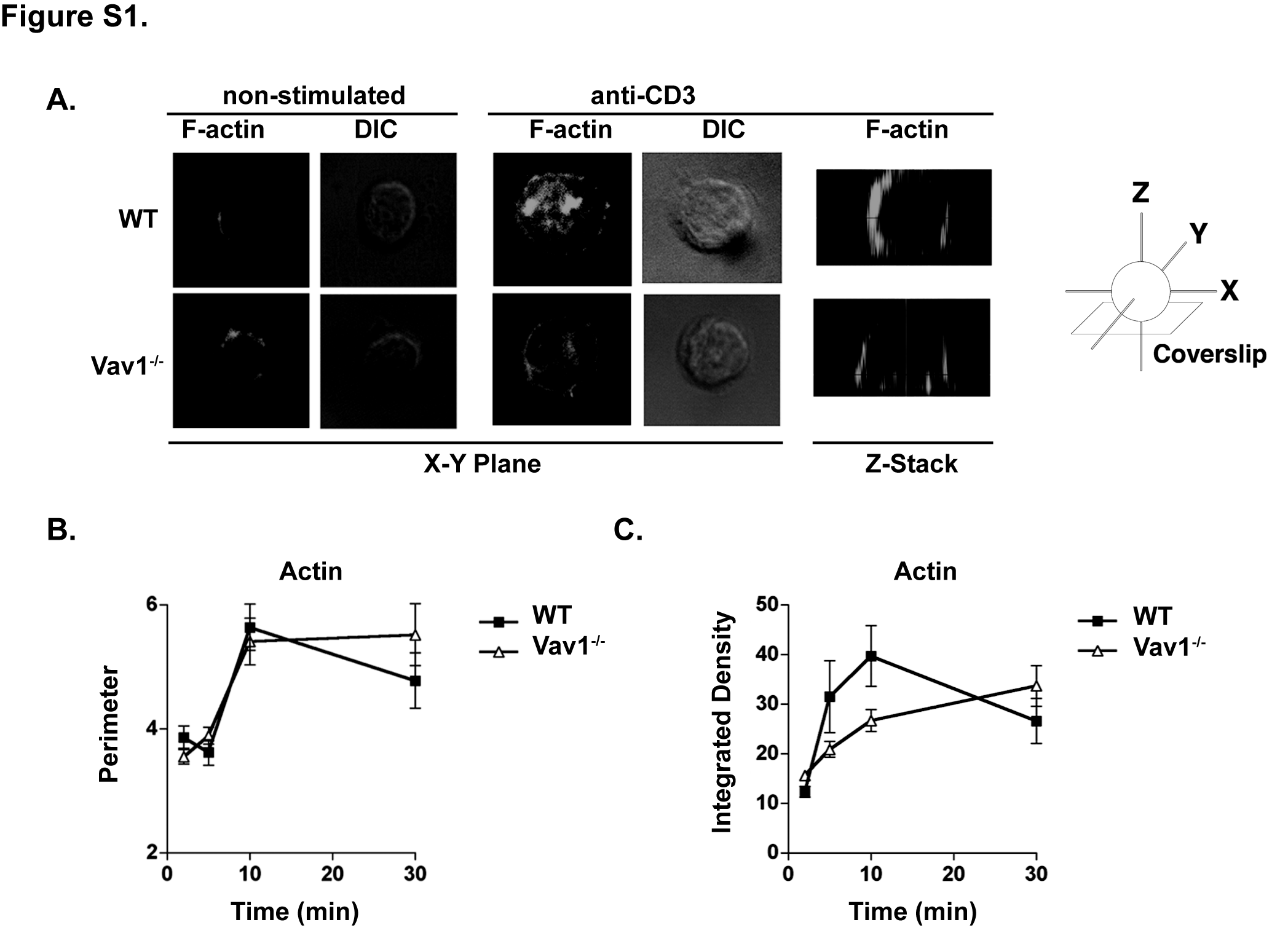

Supplement: Figure S1 — Vav1-deficient T cells show minimal defects in TCR-induced actin cytoskeletal reorganization and cell spreading. (A) Purified LN T cells from WT or Vav1−/− mice were plated on coverslips coated with anti-CD3 antibodies, and cells were subsequently stained for F-actin. Images captured by confocal microscopy depict the cell membrane-coverslip interface in the XY plane as well as Z-stacked images of the entire cell. Images shown are representative of n>10 cells for each stimulation condition. (B) T cells were stimulated and stained as in (A). Cell spreading was determined by measuring the perimeter of the membrane-coverslip interface as defined by F-actin staining. Measurements were made for n>10 cells per stimulation condition. (C) T cells from (B) were analyzed for F-actin content at the membrane-coverslip interface by measuring the pixel intensity of Alexa-Fluor-488-phalloidin fluorescence within the area defined by the perimeter of the membrane-coverslip interface (integrated density). Measurements were performed in n>10 cells per condition. (0.33 MB TIF) [file pone.0006599.s001.tif]

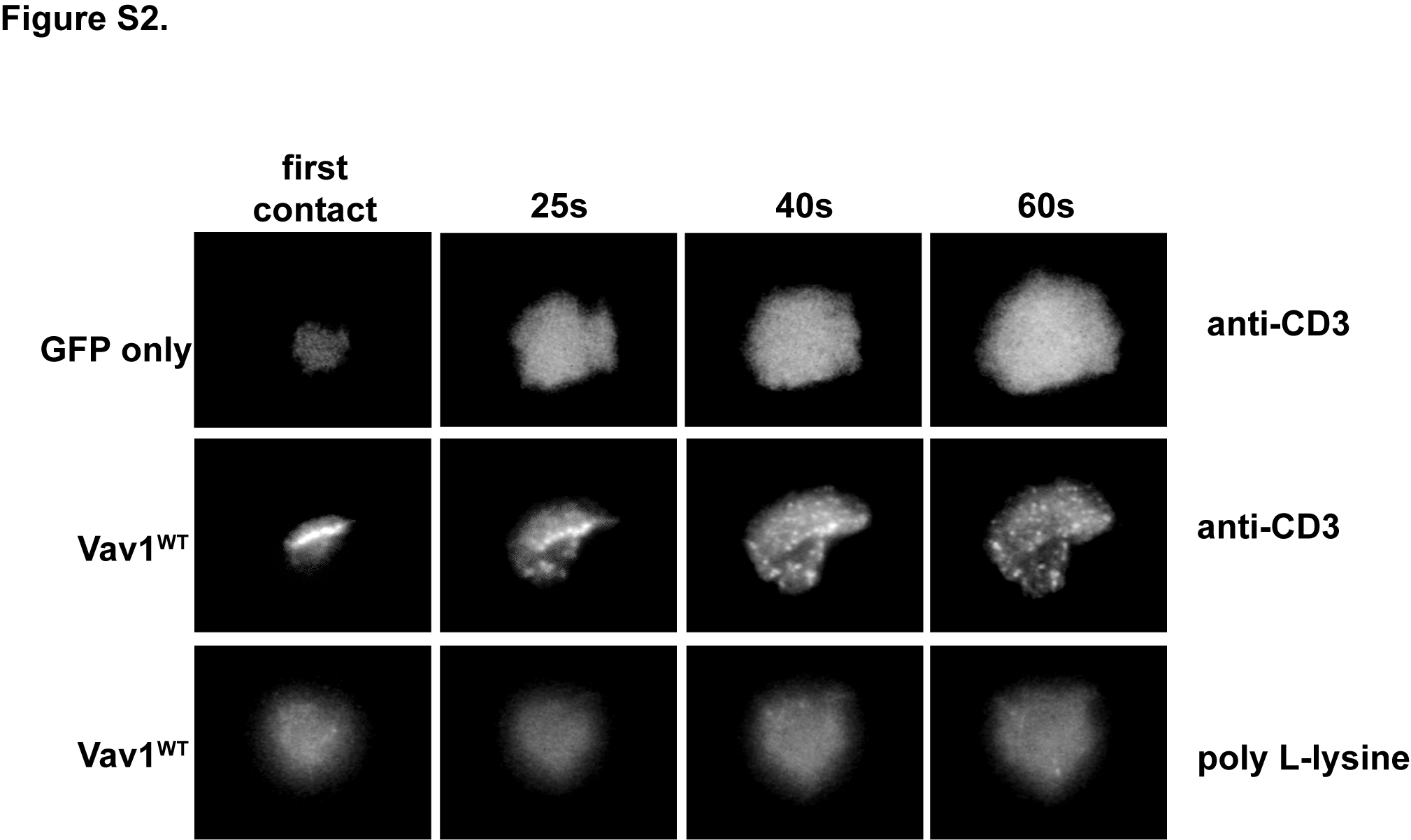

Supplement: Figure S2 — Vav1 microcluster formation is induced by TCR stimulation. Live J.Vav cells expressing GFP-only, or J.Vav1WT cells were incubated on coverslips coated with anti-CD3 antibodies, or with poly L-lysine and imaged in real time using TIRFM. (0.34 MB TIF) [file pone.0006599.s002.tif]

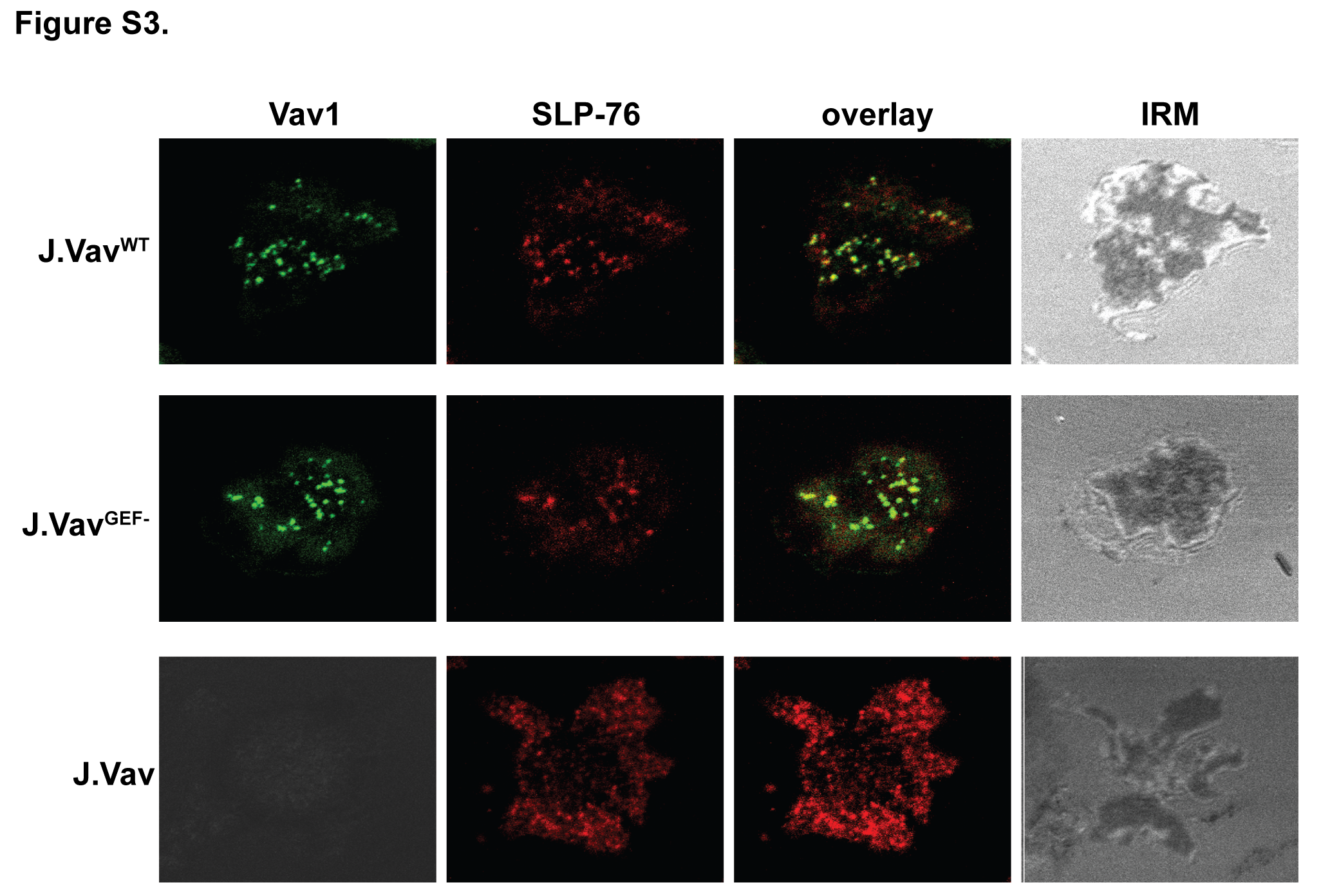

Supplement: Figure S3 — Vav1 colocalizes with SLP-76 in TCR-induced microclusters. J.Vav, J.Vav1WT, or J.Vav1GEF- cells were activated on anti-CD3-coated coverslips for 2 minutes followed by fixation and permeabilization. SLP-76 microclusters were visualized by staining with anti-SLP-76 antibodies followed by anti-rabbit-Cy5. Vav1 microclusters are GFP+. Images were captured by confocal imaging of cells within the plane of contact with the stimulatory coverslip, shown by internal reflection microscopy (IRM). Representative images are shown (n≥10). (2.27 MB TIF) [file pone.0006599.s003.tif]

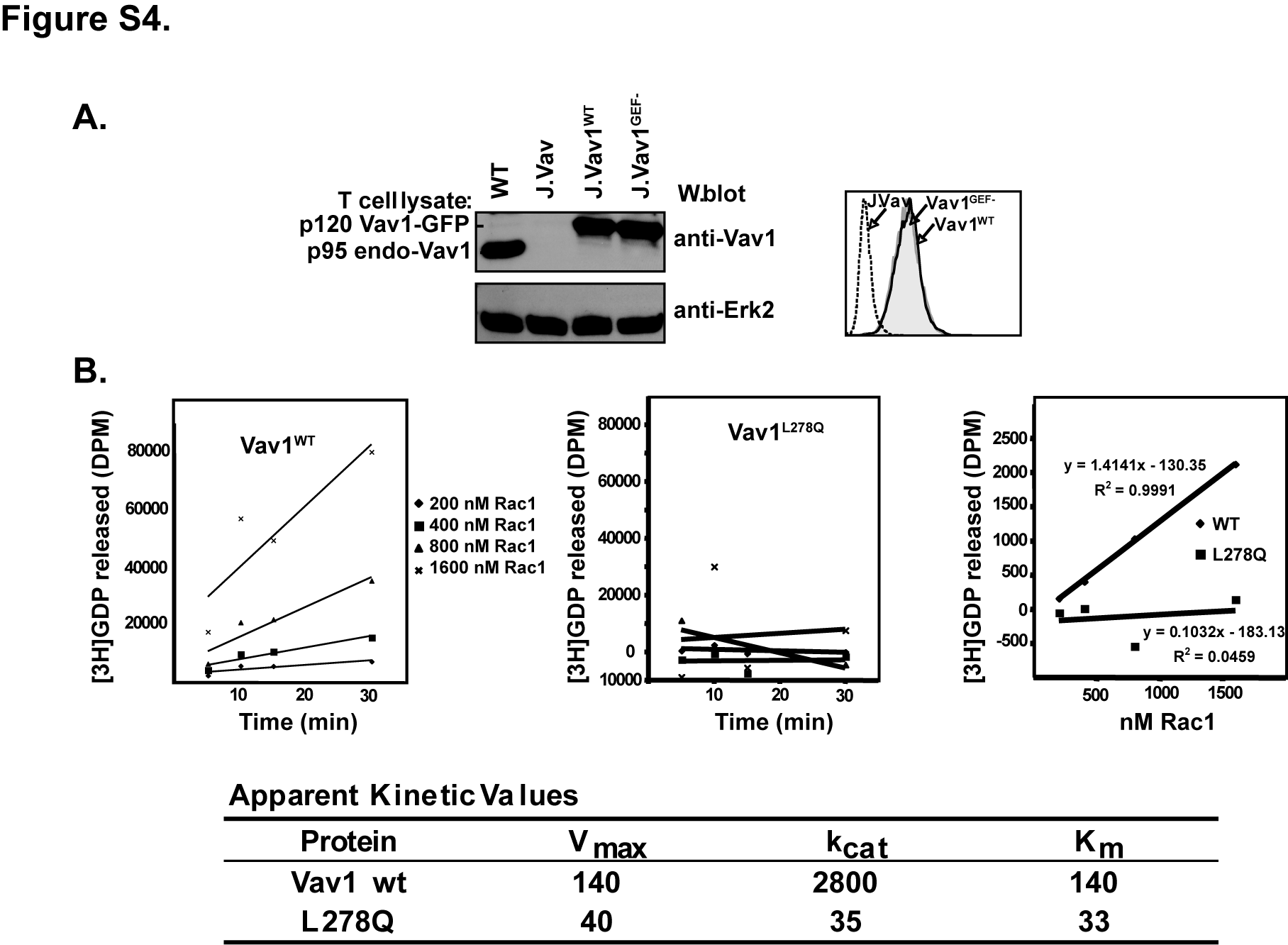

Supplement: Figure S4 — The Vav1 L278Q mutation abrogates GEF activity. (A) Stable expression of GFP-tagged Vav1WT and Vav1GEF- in J.Vav cells was similar to endogenous levels of Vav1 in Jurkats as demonstrated by immunoblotting with anti-Vav1 antibodies and by FACS. (B) (left) In vitro GDP-GTP exchange on increasing concentrations of Rac1 was measured as loss of radiolabeled [3H]-GDP in the presence of unlabeled GTP and a WT Vav1 MBP-DH-PH-ZF fusion protein or (middle) a fusion protein containing the Vav1 DH domain expressing L278Q (MBP-DH(L278Q)-PH-ZF), corresponding to L213Q in N-terminally truncated “onco” Vav, [22], [35]. (right) Kinetics of in vitro GDP-GTP exchange as shown in left and middle panels. Bottom panel: kinetic values for GDP-GTP exchange on Rac1 by WT Vav1 MBP-DH-PH-ZF or GEF-inactive MBP-DH(L278Q)-PH-ZF, as determined by Lineweaver-Burk plot shown above. (0.17 MB TIF) [file pone.0006599.s004.tif]

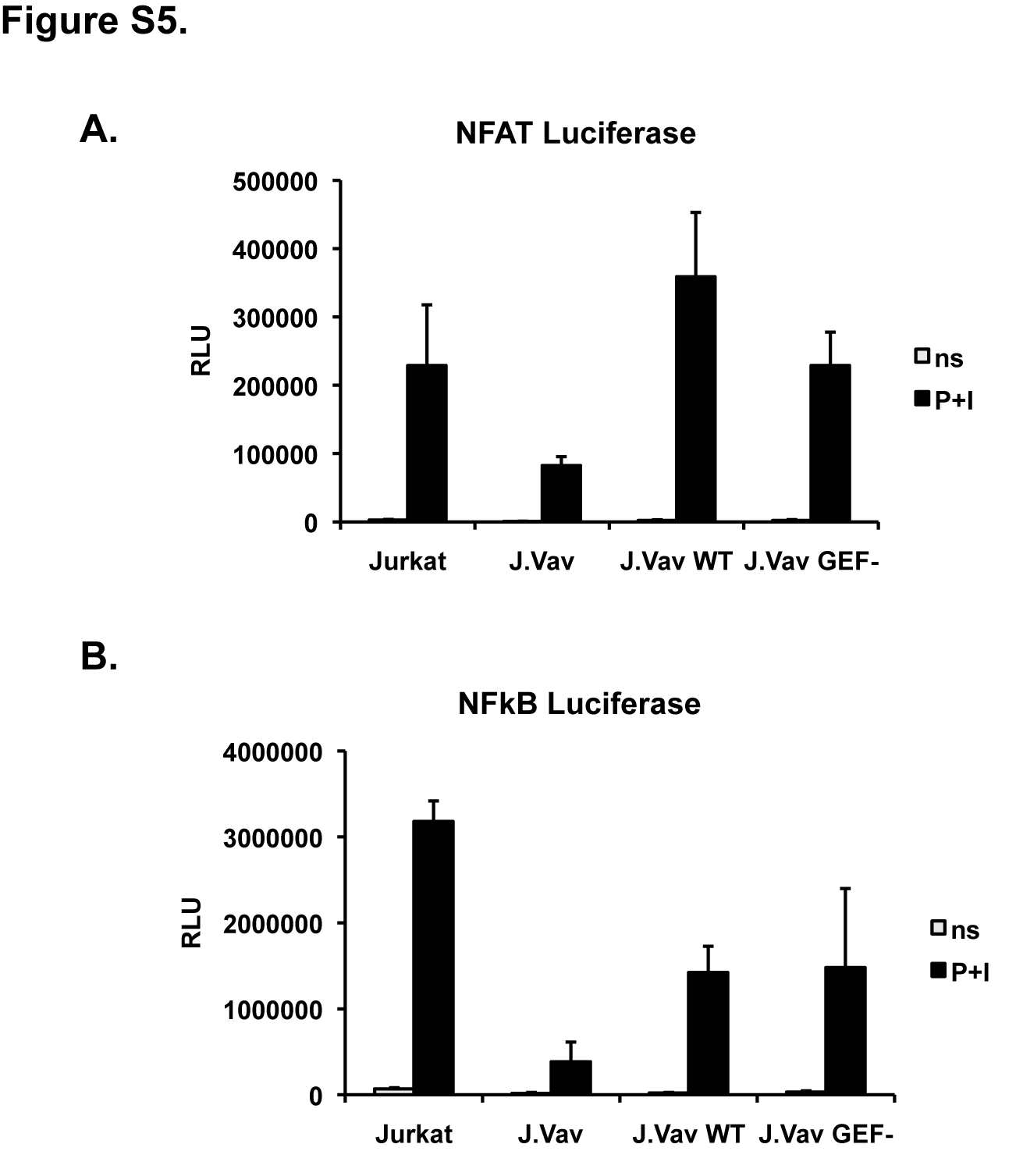

Supplement: Figure S5 — Activation of NFAT and NFkB luciferase by PMA and Ionomycin is Vav-independent. NFAT (A) or NFκB (B) luciferase reporter assays of untreated and PMA and ionomycin-activated J.Vav, J.Vav1WT and J.Vav1GEF- cells. Data are mean±SD n>5 experiments. (0.09 MB TIF) [file pone.0006599.s005.tif]
