# Supplementary material for: Vav Links the T Cell Antigen Receptor to the Actin Cytoskeleton and T Cell Activation Independently of Intrinsic Guanine Nucleotide Exchange Activity
Source: PLoS One. 2009 Aug 12;4(8):e6599. doi: 10.1371/journal.pone.0006599 (PMC2719804; doi:10.1371/journal.pone.0006599)
Supplement: Supplemental Methods S1 — (0.06 MB DOC) [file pone.0006599.s006.doc]

SUPPLEMENTAL MATERIALS AND METHODS

*J.Vav Cell Stimulation and Immunoblotting.* For stimulations and immunoprecipitations, 10 x10^6^ cells were stimulated with 1 μg/mL anti-CD3ε (clone HIT3a, BD Biosciences, San Diego, CA) + 1 μg/mL anti-IgG2a (Southern Biotechnology Associates, Birmingham, AL) for the indicated time points. Cells were washed with cold PBS and lysed in RIPA buffer (PBS, 1% NP40, 0.5% deoxycholate, 0.1% SDS, 10 mM EDTA) supplemented with a protease inhibitor cocktail (Boehringer, Ridgefield, CT), 10 mM

NaF, and 1 mM Na_3_VO_4_ for 10 minutes on ice. Crude lysates were cleared by centrifugation at 13,000 *x g* for 10 minutes at 4°C. Clarified lysates were incubated with 0.2 μg anti-Vav1 (Santa Cruz, Santa Cruz, CA) or anti-SLP-76 antibodies (Upstate, Lake Placid, NY) with rotation for 2 hours at 4°C followed by the addition of a slurry of protein A/G-conjugated Sepharose beads (Amersham Biosciences, Piscataway,

NJ) and rotation for 1 hour at 4°C. Immunoprecipitates were washed four times with cold RIPA buffer, resuspended in SDS sample buffer, and analyzed by Western blotting following standard procedures. For immunoblotting, clarified lysates were prepared as described above and resolved by SDS-PAGE followed by Western blotting with antibodies against proteins-of-interest, as indicated. Primary antibodies were developed with HRP-conjugated secondary antibodies (anti-mouse, Zymed, San Francisco, CA; anti-rabbit, Amersham Biosciences; anti-sheep, Upstate). Immune complexes were

detected by enhanced chemiluminescence (Amersham Biosciences).

*TIRFM Imaging.* Glass-bottom dishes (MatTek, Ashland, MA) were coated overnight at 4°C with 1μg/mL anti-CD3ε (clone HIT3a; BD Biosciences). Excess antibody was removed by washing 3X with non-fluorescent media (NFM: MEM plus HEPES, minus NDA, riboflavin, and folic acid). 1 x 10^6^ cells were resuspended in NFM, dropped onto coverslips and imaged using TIRF microscopy. A beam from a solid state laser (488nm, 20mW, SAPPHIRE 488-20-OPS, COHERENT, CA) was introduced into an inverted microscope (IX-81, Olympus, Japan) for illumination. Images were captured using an EB-CCD camera (C-7190-23, Hamamatsu Photonics, Japan) equipped

with an image intensifier (C8600-05, Hamamatsu Photonics, Japan). Image recording and processing were performed using AQUACOSMOS software (Hamamatsu Photonics, Japan). Image analyses were performed using MetaMorph software (Molecular Devices Corp., Sunnyvale, CA). Kymographic analysis was performed essentially as in (Yokosuka et al., 2006). In brief, an arbitrary “slice” was drawn through a cell, and was then applied to all frames of a movie using MetaMorph software. Subsequently,

the fluorescence over time of individual Vav1-GFP microclusters contained within the “slice” are visualized as white “streaks.” For analyses of fluorescence of single microclusters over time, a gate was drawn around individual, randomly chosen microclusters. This gate was applied to all frames of a movie beginning with the first frame in which the microcluster was visible, to the last frame of the movie.

Fluorescence of a selected microcluster is reflected in arbitrary units. Shown are 2 representative microclusters of n=5.

*SLP-76 Microcluster Formation.* J.Vav, J.Vav1WT, or J.Vav1GEF- cells were incubated on stimulatory anti-CD3-coated coverslips for 2 minutes. Unbound cells were gently removed with PBS and coverslips fixed in 3% paraformaldehyde at RT for 20 minutes followed by permeabilization (PBS, 2% FCS, .1% TX-100) at RT for 5 minutes. Coverslips were blocked (PBS, 2% FCS) at RT for 30 minutes followed by staining with rabbit anti-SLP-76 antibodies (Santa Cruz) at RT for 30 minutes. Coverslips were washed with blocking buffer followed by addition of anti-rabbit-Cy5 (Molecular Probles, Eugene, OR) at RT for 20 minutes. Confocal and interference reflection microscopy (IRM) images were taken using Zeiss LSM510 confocal system.

*Actin Polymerization and MTOC Polarization.* T cells were purified from LN cell suspensions by magnetic sorting and removal of B cells with anti-Ig-coated Dynabeads (Invitrogen, Carlsbad, CA) using standard procedures. Purified cells were resuspended in plain DMEM and incubated on coverslips coated with anti-CD3ε antibodies (clone 145-2C11; 1 μg/mL, BD Biosciences) or with PBS. For actin polymerization, cells were incubated on coverslips for the indicated time points. After incubation,

unbound cells were gently removed with plain DME. Cells were fixed with 37°C 2% paraformaldehyde for 20 minutes at RT followed by permeabilization PBS, 2% FCS, .1%TX-100) for 5 minutes at RT. Coverslips were blocked (PBS, 2% FCS) for 20-30 minutes at RT. Actin polymerization was visualized by staining of F-actin with AlexaFluor-488-phalloidin (Molecular Probes). Cell spreading was determined using ImageJ software to measure the perimeter of the membrane-coverslip interface in arbitrary pixel units and the relative abundance of F-actin at the membrane-coverslip interface was determined by measuring the integrated density of Alexa-Fluor-488-phalloidin fluorescence within the area confined by the aforementioned perimeter. Integrated density was determined as cumulative pixel intensity within the area constrained by the perimeter. MTOC polarization was performed by incubating purified T cells on coverslips in a 37°C tissue culture incubator for 25-30 minutes. Cells were fixed with 2% paraformaldehyde for 20 minutes at RT followed by permeabilization (PBS, 2% FCS, .1%TX-100) for 5 minutes at RT. Coverslips were blocked (PBS, 2% FCS) for 20-30 minutes at RT. MTOCs were visualized by staining with FITC-anti-α-tubulin (Sigma, St. Louis, MO) for 20 minutes at RT.

*VavNULL hematopoeitic stem cell complementation (VavNULL-HSCC).* A single dose of 150 mg/kg of 5-flurouracil (10 mg/mL in PBS, Sigma) was injected into donor mice intraperitoneally. Four to five days following injection, donors were sacrificed, femurs removed, and bone marrow (BM) flushed with DMEM supplemented with 15% FCS, pen/strep, L-glutamine, Na-pyruvate, nonessential amino acids and

2-mercaptoethanol (15% media). Collected BM was filtered through nylon mesh and red blood cells lysed in ACK buffer (Sigma). Cells were resuspended in 3-4 mL 15% media, counted, resuspended to a final concentration of 1-2 x 10^6^ cells/mL, and plated at 1 mL/well in a 24-well plate. The following cytokines were added to cultures: SCF (100 ng/mL, PeproTech, Rocky Hill, NJ), IL-3 (6 ng/mL, PeproTech), and IL-6 (10 ng/mL,

PeproTech). After 2 days in culture, the cells were retrovirally transduced via “spinfection”. First, viral supernatants were filtered through a .22 μm syringe tip filter and 1 mL filtered supernatant was added to each well with 2X cytokines and 2X polybrene (Sigma). The plate was centrifuged at 2000 rpm for 90 minutes at RT. This procedure was repeated the next day. The day of reconstitution, *rag2-/-* recipient

mice were lethally irradiated with 950 Rad (gamma irradiation (Cs^137^), MDS Nordion, Ottawa, Ontario, Canada) and allowed to rest for at least 2 hours prior to injection. Infection efficiency and viability of BM cells was assessed by flow cytometry. Cells were harvested, washed with sterile PBS, and resuspended in PBS in a volume of 250 μL x number of recipients. Mice were injected with 250 μL cell

suspension (~0.25 x10^6^ cells) invtraveinously into the tail vein using an insulin syringe and kept on antibiotic-containing water (Hi-Tech Pharmacal Co., Amityville, NY) for at least 2 weeks following injection. Four weeks post-reconstitution, PBLs isolated from recipient mice were analyzed via flow cytometry to evaluate expression of GFP-tagged Vav1 protein. Mice were sacrificed and analyzed 5-7 weeks following reconstitution.

*T Cell Stimulation and Proliferation Assays.* Purified T lymphocytes were cultured in DMEM supplemented with 10% FCS, pen/strep, L-glutamine, Na-pyruvate, nonessential amino acids and 2-mercaptoethanol (10% media). T cells were stimulated at 5 x 10^5^ cells/200 μL in 96-well U-bottom tissue culture plates with soluble anti-CD3ε antibodies (clone 145-2C11, 1 μg/mL, BD Biosciences) +/- anti-CD28 (clone 37.51, 5 μg/mL, BD Biosciences), or SEE (Toxin Technologies, Sarasota, FL) as indicated, at 37°C. Cells were pulsed with 1 μCi [^3^H]-thymidine at 48 hours for an additional 12-16 hours and then

collected and analyzed in a scintillation counter. All assays were conducted in triplicates. The data are displayed as raw cpm values. For CFSE labeling, cells were washed in PBS and labeled with 1 μM CFSE (Molecular Probes) for 15 minutes at 37°C. Cells were washed with 10% media and stimulated as indicated for 72 hrs. Cells were stained with anti-CD4-APC conjugates (BD Biosciences) and proliferation was analyzed

by flow cytometry.

*T Cell Polarization and Analysis of Cytokine Production.* Naïve CD4+CD62L+ LN T cells were FACS sorted and stimulated in 7-day cycles, starting with stimulation on day 0, followed by expansion on day 3 and the collection of resting cells on day 7. For primary stimulation, purified naïve T cells were activated with 1 μg/mL anti-CD3 (clone 145-2C11, BD Biosciences) + anti-CD28 antibodies (clone 37.51, BD Biosciences) and irradiated (2,000 Rad) C57/B6 *rag2-/-* splenic APCs, in the presence of heat-killed

*Listeria monocytogenes* (a gift from Dr. E. Unanue) and 10 μg /mL of anti−IL-4 (11B11, a gift from Dr. K. Murphy) for Th1 differentiation, and 100 U/mL of IL-4 (a gift from Dr. K. Murphy) and 3 μg/mL of anti-IL-12 (TOSH, a gift from Dr. K. Murphy) for Th2 differentiation. On day 3, cells were expanded in fresh media containing 40 U/mL of IL-2. On day 7, cells were restimulated with antibodies and irradiated C57/B6 *rag2-/-* splenic APCs in the presence of either Th1 or Th2 cocktails. For ELISA, resting cells

were stimulated with anti-CD3 antibodies + cross-linking for 24 hours. Culture supernatants were collected and cytokine concentrations were measured using Cytometric Bead Array (BD Biosciences) according to manufacturer’s instructions.

*Ca^++^ Fluxes.* Ca^++^ signaling was measured by preloading total LN cell suspensions with Fluo-4-AM (Molecular Probes) at a concentration of 3-5 μg/mL, 2 x 10^6^ cells/mL for 45 minutes at 37°C with occasional vortexing. Cells were stained with anti-CD4-APC conjugates (BD Biosciences) after loading to allow gating on CD4+ T cells during analyses. Cells were washed and resuspended in 10% media and analyzed by flow cytometry by running unstimulated samples for 10 seconds to establish baseline. Cells

were stimulated by addition of 1 μg/mL anti-CD3 (clone 145-2C11, BD Biosciences) immediately followed by cross-linking with anti-hamster antibodies (Jackson Immunolaboratories, West Grove, PA). Ca++ fluxes were measured for 7 minutes followed by the addition of ionomycin for an additional 1 min at 0.5 μg/mL.

*Rac assay.* Purified LN T cells were starved for 30 minutes in media lacking serum. Cells were stimulated with 1 μg/mL anti-CD3 (clone 145-2C11, BD Biosciences) + anti-hamster antibodies (Jackson Immunolaboratories) for 2 minutes and Rac assay performed using EZ-Detect Rac1 Activation Kit (Pierce, Rockford, IL) according to manufacturer’s instructions. In short, cells were lysed and clarified supernatants were incubated with PBD-GST fusion protein and glutathione beads at 4°C for 1 hour with rotation. Following incubation, beads were centrifuged at 7200 *x g* for 1 minute and washed with lysis buffer. Washing was repeated two more times. SDS loading buffer was added to samples followed by boiling for 5 minutes and then spinning at 7200 *x g* for 2 minutes. Samples were then run on a 10% SDS acrylamide gel and immunoblotted for Rac1, according to standard procedures.

*Purification of GST-Rac1 and MBP-Vav1.*  An MBP-Vav1 expression vector encoding amino acids 191-578 of human Vav1 was obtained by PCR amplification. The fragment was inserted into the *Bam*HI and *Hind*III sites of pMAL-2c (New England Biolabs, Ipswich, MA) using standard cloning techniques. The L278Q mutant was generated using the Statagene Quikchange kit (Stratagene, La Jolla, CA) and the manufacturer’s mutagenesis protocol. MBP fusion proteins were expressed in E. coli strain BL21(DE) by culturing at 37°C for 3 hours and inducing protein expression with 0.05 mM isopropyl-β-D-thiogalactaside for 8 hours at 18°C. The fusion protein was purified using amylose resin using the manufacture’s protocol, with the exception that the column was washed with 20 mM Tris, pH7.4, 200 mM NaCl after binding the protein to the resin. The MBP-fusion proteins were eluted with the same buffer containing 10 mM

maltose.

*Guanine Nucleotide Exchange Assays.* For each individual assay, 25 pmol of GST-Rac1 was loaded with labeled GDP in a buffer containing 20 mM Tris, pH 8.0, 3 mM MgCl_2_, 10 mM EDTA, 1 mM DTT, and 0.2 μM [^3^H]GDP for 20 min at 37°C. Following the incubation, 375 mM MgCl_2_ was added to a final concentration of 20 mM and the mixture

incubated at room temperature for 5 min. The [^3^H]GDP loaded Rac1 was then diluted 3-fold in buffer containing 20 mM Tris pH 8.0, 100 mM NaCl, and MBP-Vav at the indicated concentrations. The mixture was allowed to incubate for 10 min. before a 250-fold excess of unlabeled GTP was added. Aliquots were removed at indicated time points, diluted in 1 ml of ice-cold stop buffer (20 mM Tris, pH 7.4, 150 mM NaCl, and 25 mM MgCl_2_), and passed through nitrocellulose filters. The filters were washed with 4 mL of stop buffer, dried, and the bound [^3^H]GDP was counted via liquid scintillation

counting.
